# Phylogeographic Patterns in Africa and High Resolution Delineation of Genetic Clades in the Lion (*Panthera leo*)

**DOI:** 10.1038/srep30807

**Published:** 2016-08-04

**Authors:** L. D. Bertola, H. Jongbloed, K. J. van der Gaag, P. de Knijff, N. Yamaguchi, H. Hooghiemstra, H. Bauer, P. Henschel, P. A. White, C. A. Driscoll, T. Tende, U. Ottosson, Y. Saidu, K. Vrieling, H. H. de Iongh

**Affiliations:** 1 Leiden University, Institute of Environmental Sciences (CML), PO Box 9518, 2300 RA Leiden, The Netherlands; 2 Leiden University, Institute of Biology Leiden (IBL), PO Box 9505, 2300 RA Leiden, The Netherlands; 3 Forensic Laboratory for DNA Research, Department of Human Genetics, Leiden University Medical Centre, P.O. Box 9600, 2300 RC Leiden, The Netherlands; 4 Qatar University, Department of Biological and Environmental Sciences, College of Arts and Sciences, PO Box 2713, Doha, Qatar; 5 Institute for Biodiversity and Ecosystem Dynamics, University of Amsterdam, Science Park 904, 1018 XH Amsterdam, The Netherlands; 6 WildCRU, Recanati-Kaplan Centre, University of Oxford. Tubney House, Abingdon Road, OX13 5QL, UK; 7 Panthera, 8 West 40th Street, 18th Floor, New York, NY 10018, USA; 8 Center for Tropical Research, Institute of the Environment and Sustainability, University of California, Los Angeles, CA 90095-1496, USA; 9 Wildlife Institute of India, Dehradun 248001, Uttarakhand, India; 10 A. P. Leventis Ornithological Research Institute, P.O. Box 13404 Jos, Nigeria; 11 Nigeria National Park Service, PMB 0258 Garki-Abuja, Nigeria; 12 University of Antwerp, Department Biology, Evolutionary Ecology Group, Groenenborgerlaan 171, 2020 Antwerpen, Belgium

## Abstract

Comparative phylogeography of African savannah mammals shows a congruent pattern in which populations in West/Central Africa are distinct from populations in East/Southern Africa. However, for the lion, all African populations are currently classified as a single subspecies (*Panthera leo leo*), while the only remaining population in Asia is considered to be distinct (*Panthera leo persica*). This distinction is disputed both by morphological and genetic data. In this study we introduce the lion as a model for African phylogeography. Analyses of mtDNA sequences reveal six supported clades and a strongly supported ancestral dichotomy with northern populations (West Africa, Central Africa, North Africa/Asia) on one branch, and southern populations (North East Africa, East/Southern Africa and South West Africa) on the other. We review taxonomies and phylogenies of other large savannah mammals, illustrating that similar clades are found in other species. The described phylogeographic pattern is considered in relation to large scale environmental changes in Africa over the past 300,000 years, attributable to climate. Refugial areas, predicted by climate envelope models, further confirm the observed pattern. We support the revision of current lion taxonomy, as recognition of a northern and a southern subspecies is more parsimonious with the evolutionary history of the lion.

Insight into the phylogeographic patterns of co-distributed taxonomic groups may lead to previously unrecognized biogeographic patterns, a greater understanding of evolutionary histories, and contribute to guiding conservation decisions^1^,^2^,^3^. On the African continent we observe strongly congruent phylogenetic patterns for savannah mammals with a comparable, continent-wide distribution. The distribution of subspecies and species complexes tends to follow a north-south axis in sub-Saharan Africa, with West and Central Africa populated by sister taxa different from those found in East and Southern Africa^4^,^5^ (regions defined following the lion conservation strategies^6^,^7^). This north-south dichotomy is further confirmed by genetic data from numerous ungulates and carnivores (Fig. 1)^8^,^9^,^10^,^11^,^12^,^13^. For most species, distinct subspecies are recognized, while the African lion (*Panthera leo leo*) is currently considered as a single subspecies.

Phylogenetic data of lion populations indicate that current taxonomy does not sufficiently reflect the genetic diversity within the African lion^14^,^15^,^16^,^17^,^18^,^19^,^20^,^21^. Notably, lion populations from West and Central Africa have a distinct phylogenetic position, with a nested position of the Asiatic subspecies (*P*. *leo persica*)^14^,^15^,^19^,^21^. The validity of the subspecies status of the Asiatic lion, nowadays confined to a single population in India, is thereby challenged. Previous studies lacked comprehensive sampling of the West and Central African region and based their results on relatively small sample sizes^14^,^15^,^16^,^17^,^19^,^21^. As a consequence, the position of West and Central African lion populations and their relation to the Asiatic subspecies in the phylogenetic tree remained largely unresolved^14^,^19^,^21^.

The present study aims to provide a more complete overview of genetic diversity within the African lion and compares results to phylogeographic patterns and taxonomy in a range of African savannah mammals. In addition, we estimate the dates of the major splits in the phylogenetic tree and relate the observed patterns to the dynamic climate history of Africa. Discordances between phylogeographic patterns derived from mtDNA and nuclear loci have been reported in a limited number of species^22^,^23^. However, previous studies on lions have shown that mitochondrial DNA (mtDNA) loci produce phylogenies that are compatible with phylogenies based on autosomal data^18^,^24^, supporting the use of mtDNA as an appropriate marker for the current study. For sixteen lions the complete mitochondrial genome was analysed; 1454 base pairs (bp) of the mtDNA were analysed for additional 178 lions throughout the complete geographic range (see Fig. 2 and Supplemental Table 1 for sampling locations). This included samples from each of the Lion Conservation Units (LCUs) in West and Central Africa in which the persistence of lion populations have recently been confirmed^25^,^26^. To reconstruct the evolutionary history of the West and Central African lion, museum samples from extinct populations in North Africa and Asia, representing a historical connection between the African and the Asiatic subspecies, were obtained and processed using an approach suitable for ancient DNA (aDNA).

This is the first study in which phylogeographic patterns of a large number of savannah mammals from different trophic levels is compared and put into a context of climatic changes over the past 300,000 years (300 kyr). We have assessed inter and intraspecific distinctions for all orders of large mammals with a pan-African distribution (the orders Chiroptera, Insectivora, Lagomorpha and Rodentia were excluded). As a model taxon, the lion is used to generate a high resolution map of the distribution of haplogroups. The results contribute to a better understanding of the evolutionary forces that shaped the phylogenetic patterns observed among numerous savannah mammals on the African continent, including humans^27^. Results should be translated into recommendations for the management of different populations and species. In light of our findings, we challenge current lion taxonomy that recognizes only the African and the Asian subspecies, and we investigate options for a taxonomic revision that is more parsimonious with the newly revealed evolutionary history of the lion.

## Results

Bayesian, Maximum Likelihood (ML) and Maximum Parsimony (MP) trees were constructed from three different alignments: 1) cytochrome b and control region (hereafter cytB + ctrl reg.), 2) the complete mitogenome, and 3) a combination of both datasets. All showed identical topology, and trees including the complete mitogenome showed strongly significant support for a basal split separating lions in the northern part of their range (North group: West Africa, Central Africa, and North Africa/Asia) and lions in the southern part of their range (South group: North East, East/Southern, and South West Africa) (Fig. 3a; mitogenome tree shown in Supplemental Fig. 1). Within the North group, a clade that included all Asiatic lions and aDNA sequences from North Africa and Iran was significantly supported, as was a clade with Central African lions and the clade with West African lions. Lions from Central Africa and the North Africa/Asia clade are grouped together on a well-supported branch. In the South group, three major groups can be distinguished: a South West group, an East/Southern group and a North East group. All three clades are significantly supported, as is the branch combining the East/Southern and North East group. The same structure can be seen in the haplotype network based on cytB + ctrl reg. (Fig. 4). The observed groups are indicated together with the sample location in Fig. 2. In only two cases did we observe haplotypes from distinct phylogenetic groups in the same geographic region: in Ethiopia we found haplotypes from the Central Africa group as well as from the North East group, and in the Republic of South Africa (RSA) we found haplotypes from East/Southern and the South West group (Fig. 2, shaded areas).

Analysis of diversity indices for each of the main phylogenetic groups indicate that the East/Southern Africa clade is least diverse, both in terms of number of haplotypes and distance between haplotypes (Supplemental Table 2). The South West Africa clade is most diverse with notably many differences between haplotypes. Further it shows that pairwise differences within the North group (i.e. haplogroups West Africa, Central Africa, and North Africa/Asia), are relatively few, compared to the South group, or in comparison to differences between the North and South group. Population size is estimated to have been constant throughout the past 300 kyr, following from a Bayesian Skyline plot.

The most recent common ancestor of all modern lions was estimated to have existed around 245,000 years before present (245 ka) (95% Highest Posterior Density (HPD): 120–385 ka; ESS: 216). The split of the South group is older than the North group, estimated to be 189 ka (95% HPD: 90–300 ka; ESS: 253) and 142 ka (95% HPD: 60–239 ka; ESS: 273), respectively. For all major clades (nodes of all haplogroups ESS > 280) the date of the most recent common ancestor was estimated and compared to results from previous publications (Supplemental Table 3, Fig. 3a,b).

## Discussion

In this paper, we describe the phylogeographic patterns shared among several orders of African savannah mammals, and examine the phylogenetic relationships of lion populations throughout their entire geographic range. We have analysed 194 lion sequences of cytB + ctrl reg., including 30 aDNA sequences and 16 complete mitochondrial genomes. This approach has produced strong support for a basal dichotomy between lion populations from the northern part of their range and those from the southern part. Within the basal dichotomy, six major phylogenetic groups are identified: West Africa, Central Africa and North Africa/Asia (North group) and North East, East/Southern and South West (South group). This study included samples from 22 countries, including all LCUs with a confirmed lion population in West and Central Africa, as well as extinct populations, representing a comprehensive overview of the historical geographic range of the modern lion. All zoo and museum samples used in our study included decisive information on the origin of the individual or its free-ranging ancestors (for additional information see Supplemental Information 1).

Based on the available datapoints, a proposed range of the haplogroups was generated, showing two areas of admixture between distinct lineages (Fig. 2). Although the Rift Valley has been proposed as a barrier for gene flow in lions^14^,^15^,^16^,^19^,^28^,^29^, our denser sampling of the region between the North and South groups found that the Rift Valley does not completely prevent a mixture of haplotypes between the two basal branches in the phylogenetic tree (haplotypes 9, 12–14). The second admixture zone is located around Kruger National Park (NP) and Limpopo-Venetia National Reserve (NR), RSA, in which we detect haplotypes from the South West group (haplotype 20 and 22) in addition to haplotypes from the East/Southern group (haplotype 15). Since lions from other parts of RSA and the southern range of Botswana and Namibia also cluster to the East/Southern group, it is likely that the mixture of haplotypes in the Kruger/Limpopo area is the result of human-induced translocations. Lions from Etosha have frequently been used in translocations to South Africa. Further, some private reserves adjacent to Kruger NP that were initially fenced off, are now connected to the park^30^.

**Figure 1:**
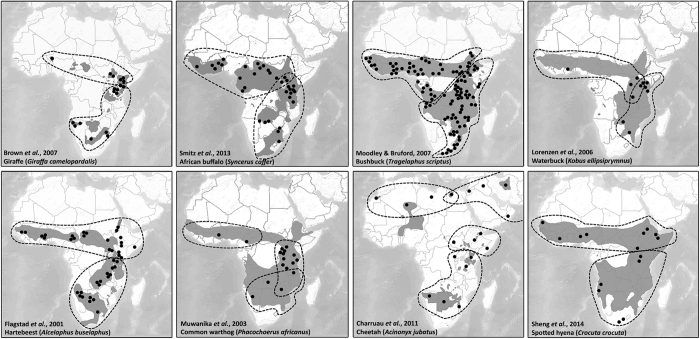
Examples from eight species for which a dichotomy between West/Central African populations and populations in East/Southern Africa has been shown in phylogenetic data: giraffe (*Giraffa camelopardalis*), African buffalo (*Syncerus caffer*), bushbuck (*Tragelaphus scriptus*), waterbuck (*Kobus ellipsiprymnus*), hartebeest (*Alcelaphus buselaphus*), common warthog (*Phacochoerus africanus*), cheetah (*Acinonyx jubatus*) and spotted hyena (*Crocuta crocuta*). Species were selected from different orders and based on available phylogenetic data which resulted in well-resolved trees. Sample locations (black dots) are indications and are not necessarily proportional to the number of collected samples. The most basal phylogenetic groups identified are delineated. Basemap with range data are from IUCN^80^.

The pattern we describe for the lion is highly congruent with phylogeographic data from different taxonomic groups occupying a range of trophic levels, implicating the environment as an evolutionary driver. The most basal dichotomy, distinguishing a northern and a southern lineage, is found in numerous savannah mammals, which we briefly review in Table 1. Several phylogeographic studies on African savannah mammals have described three main clades: West/Central Africa, East Africa and Southern Africa, suggesting that there may have been important refugial areas in these regions during the more recent part of the Pleistocene climatic cycles^4^,^5^. These three clades are clearly distinguishable in the lion based on mtDNA (nodes c, d and g in Fig. 3a) and autosomal data^24^. A model-based study on the habitat suitability for mammals and birds during the last glacial maximum (LGM) suggests the existence of five possible refugia in sub-Saharan Africa: Upper Guinea, Cameroon Highlands – Congo Basin, Ethiopian Highlands, Angola-Namibia, and East/Southern Africa^31^. These areas coincide with the five sub-Saharan lion groups, described in this study as West, Central, North East, South West, and East/Southern Africa, respectively. In addition to the most basal dichotomy, shown for other species in Table 1 and Fig. 1, the South West clade, which harbors lion populations from Angola and Namibia, is also represented in giraffe (*Giraffa camelopardalis*)^9^,^32^, zebra (*Equus zebra*)^33^, impala (*Aepyceros melampus*)^34^, greater kudu (*Tragelaphus strepsiceros*)^34^ and sable antelope (*Hippotragus niger*)^35^. Within East Africa, the North East clade is also found in kob (*Kobus kob*)^36^, oryx (*Oryx beisa*)^37^, impala (*Aepyceros melampus*)^34^ and greater kudu (*Tragelaphus strepsiceros*)^34^. Finally, the distinction we find between the West and the Central African lion is also seen in the phylogeographic pattern of roan antelope (*Hippotragus equinus*)^38^, potentially as a result of the lower Niger River acting as a permanent barrier for gene flow in these species. Climatological events have also heavily influenced migration of early humans^39^,^40^ and as a result, similar major clades and phylogeographic patterns are found in human datasets^41^,^42^,^43^.

Phylogenetic variation within the six geographic groups of the modern lion appears to have mainly emerged within the last c. 100 kyr, including the cool last glacial period (Marine Oxygen Isotope Stage (MIS) 4, 3 and 2) and two warmer periods (MIS 5 and 1)^44^,^45^. Phylogenetic structure that had evolved in regional lineages during the previous glacial-interglacial cycles, mostly disappeared by c. 100 ka attributable through various events, including genetic bottlenecks involving expansions and contractions from/to regional refugia^31^,^46^,^47^. Since the HPD intervals are relatively large, we add a palaeoclimatic context in order to propose a possible scenario that has contributed to the current phylogeographic pattern in the lion and in other sub-Saharan mammals. The two major vegetation zones on the African continent that likely influenced lion distribution through exclusion are dry desert and dense rain forests^48^,^49^, representing both hydrological extremes. In the tropics, the hydrological cycle that results in barriers and connective zones for lion dispersal, is mainly driven by latitudinal migrations of the intertropical convergence zone (ITCZ), associated with the 21 kyr precession cycle of orbital climate forcing. This 21 kyr precession cycle occurs five times within a c. 100 kyr eccentricity cycle and equates to a full interglacial-glacial period^45^,^50^. The last coalescence between the North and South lineage (node a in Fig. 3a) in the lion is estimated at ~245 ka, positioned at the start of interglacial MIS 7 (243–191 ka) (ages after Imbrie *et al.*^51^). Following the southward expansion of the Sahara, the last coalescence is characterized by a maximum monsoon index that allowed the dense wet forest to expand maximally northwards along an east-west axis in lower latitude Africa^46^,^52^,^53^,^54^,^55^,^56^,^57^. Such a vegetation pattern likely reduced or possibly eliminated the connection between northern and southern lion populations. Other species show similar dates for divergence between northern and southern populations, e.g. baboon^58^, giraffe^9^ and hyena^12^,^59^, although broader 90–95% confidence intervals overlap in the case of wild cat^60^ and cheetah^13^. It is likely that the cyclic character of orbital forcing, and the concomitant distribution of rain forest and desert in Africa repeatedly created isolated refugia and suture zones for lions and co-distributed species.

The second oldest split between South West group and East/Southern & North East groups (node b in Fig. 3a) occurred at around ~189 ka, a moment positioned at the end of interglacial MIS 7 and the transition to the first cool interval of the following glacial MIS 6. During this interval the monsoon index was still high^57^ and an extension of the Zambesian rain forest may have presented a barrier to lion populations in the South West. Simultaneously, lions belonging to the East/Southern group were distributed in a large area across East and Southern Africa^14^,^55^. More recent radiation of the South West group (node g in Fig. 3a), estimated to have occurred ~92 ka, coincides with vegetation change in the Early Glacial (MIS 5.2), following a period of droughts in which suitable habitat was reduced in Southern Africa, notably in the Kalahari region^61^. The splits between East/Southern and North East Africa (node c in Fig. 3a), located in present-day Kenya, and between West Africa and Central & North Africa/Asia (node d in Fig. 3a), located in present-day Nigeria, appear to have occurred during MIS 6 (186–128 ka) when two periods of dry and cool conditions prevailed^57^,^62^. The splits in the North group are likely due to the periodically maximum north-south extension of the Sahara desert^14^,^47^,^55^,^57^,^63^,^64^,^65^. A connection between the North Africa/Asia group and the Central Africa group may have persisted during the short periods in which the monsoon front reached high latitudes, explaining the close genetic relationship of the Central African populations to the North Africa/Asia clade^57^,^64^,^65^. The West African population possibly became isolated and reduced in numbers by the significant southwards expansion of the Sahara during MIS 4 (71–59 ka)^40^,^57^,^61^,^64^,^65^, with radiation beginning around 50 ka. There are no indications from our data that the current lion population in India was sourced or reinforced by introductions from sub-Saharan African lions, as was recently hypothesized^66^.

The estimates for the time to the most recent common ancestor (TMRCA) presented in this study deviate from the results from Barnett *et al.*^21^. Exclusion of taxa or truncation of sequences, as well as different settings for the runs (prior settings, used substitution model and substitution rate), did not sufficiently explain the observed deviation. Antunes *et al.*^18^ used an alternative approach to calculate a substitution rate, resulting in estimates for which the 95% CIs completely overlap with our 95% CIs for all but one split, which has an overlap of 63% (Supplemental Table 3).

The deep ancestral split within the African lion and the topology of the phylogenetic tree, along with the nested position for the Asiatic subspecies, clearly illustrate and support the contention that the current taxonomic division does not reflect the evolutionary history of the lion. Consequentially, it hampers priority setting for lion conservation, particularly in West and Central Africa. Because the distinct genetic lineages within the African lion are further supported by nuclear data^18^,^24^ and morphological data^67^,^68^, we suggest recognizing a northern subspecies, including West Africa, Central Africa and North Africa/Asia, and a southern subspecies, including the North East, East/Southern and South West lineages, in line with the proposed revision by Barnett *et al.*^21^. In the absence of conflicting conclusions based on other genetic markers, the distinct phylogeographic clades within the proposed two subspecies should be managed as Evolutionary Significant Units (ESUs), sensu Moritz^69^. Data from more nuclear loci, and from sampling locations at the geographical borders of the proposed haplogroup ranges may provide additional insight, but are not likely to change the main pattern described in this paper.

Our study shows a fine-scale phylogeographic pattern for the lion, with strongly significant support for a basal north-south dichotomy, as is also observed in other African savannah mammals. By analysing samples from a larger range of localities, the phylogenetic position of the Asiatic subspecies was resolved and it was possible to propose ranges and connectivity zones for six major phylogenetic clades: West Africa, Central Africa and North Africa/Asia (North group) and North East, East/Southern and South West (South group). In the context of the presented time estimates, our results contribute to understanding the evolutionary forces that shaped the genetic make-up of several African savannah mammals, and of the African lion in particular.

## Materials and Methods

**Figure 2:**
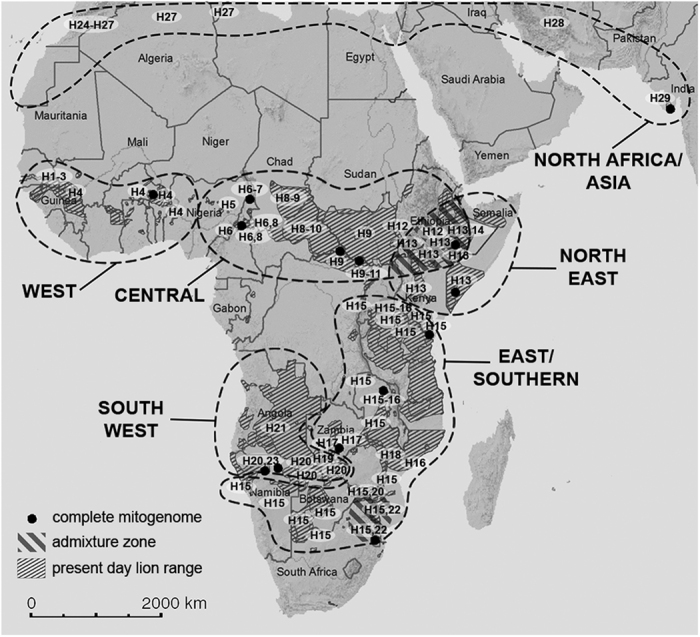
Locations of lion samples and haplotype numbers included in this study. Proposed phylogenetic lineages are delineated with dashed lines. Admixture zones in which haplotypes from different phylogenetic lineages are found are indicated by shading. Basemap with range data are from IUCN^80^.

**Figure 3:**
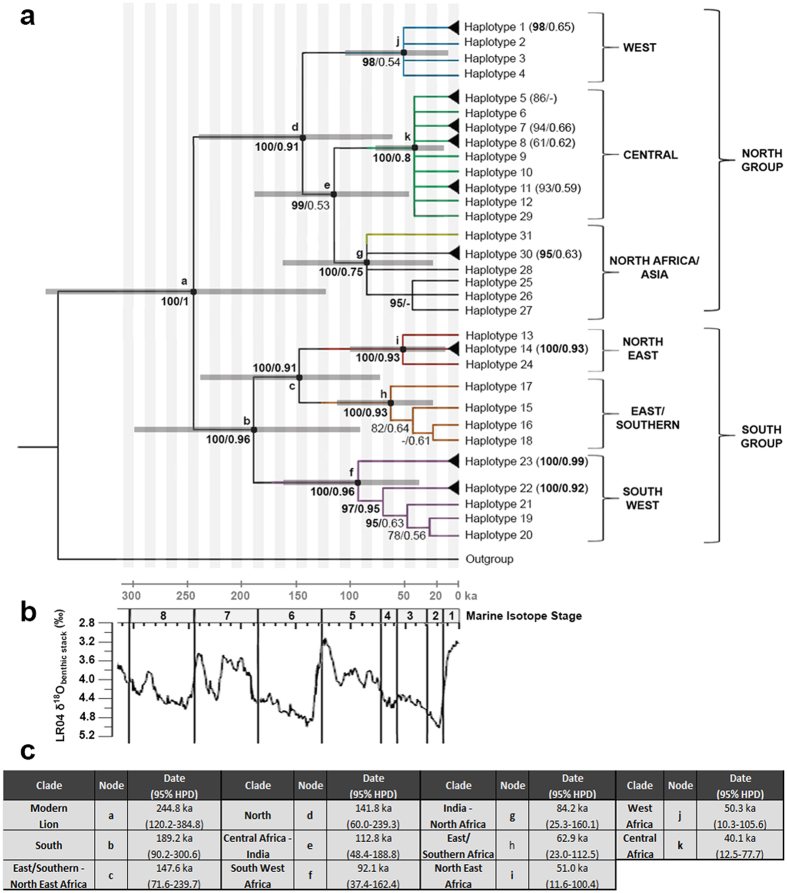
Phylogenetic analyses for the complete lion dataset, including sixteen mitochondrial genomes and 175 cytb + ctrl reg. sequences. (**a**) Phylogenetic tree of lion populations throughout their complete geographic range, based on complete mitochondrial genomes and cytB + ctrl reg. sequences. Branch colours correspond to haplotype colours in Fig. 4. Support is indicated as posterior probability (Bayesian analysis)/bootstrap support (ML analysis). Branches with a single haplotype have been collapsed to improve readability. Support for these branches is indicated by a black triangle at the tip of the branch (support shown in the label). Nodes which have been included for divergence time estimates are indicated with letters and 95% HPD node bars. Distance to outgroup and nodes without dated splits are not in proportion to divergence time. (**b**) Global oxygen isotope (∂^18^O) record showing two full interglacial-glacial cycles 7–6 and 5–2 (each of ca. 100 kyr duration), and the present interglacial 1, mainly related to global reorganisations of ocean and atmospheric temperature. In the African (sub)tropics temperature amplitude is lower than expressed in this (global) graph, however precipitation changes are higher than in the temperate and arctic areas and occur in 21 kyr precession cycles. Five maxima at ca. 21 kyr distance in time can be identified in each full interglacial-glacial cycle; precipitation maxima do not necessarily coincide with these temperature maxima^81^. (**c**) Divergence estimates in thousand years ago (ka) and 95% HPD from BEAST analysis, also indicated as error bars in Fig. 3a.

**Figure 4:**
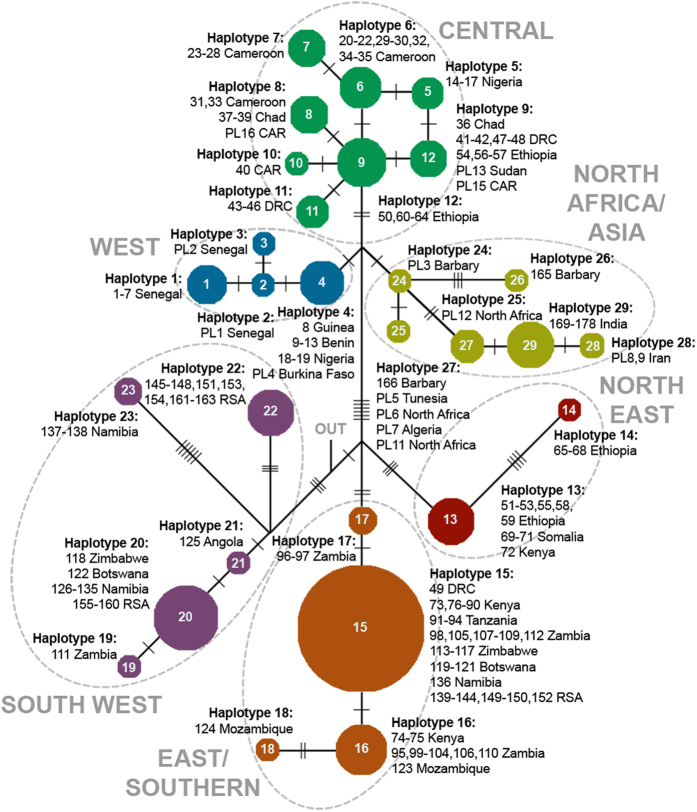
Haplotype network based on cytB + ctrl reg. sequences of lions throughout their entire geographic range. Dashed lines indicate the groups discerned by Bayesian/ML analysis in Fig. 3a. Haplotype size is proportional to its frequency in the dataset. Hatch marks represent a change in the DNA sequence. The connection to outgroup species is indicated by “OUT”.

A total of 194 samples from lions of 22 different countries were analysed, including samples previously described in Bertola *et al.*^19^,^24^ and Barnett *et al.*^21^ (Supplemental Table 1 and Fig. 2). Blood, tissue or scat samples were collected from free-ranging individuals or captive lions with proper documentation of their breeding history, in accordance with relevant guidelines and in full compliance with specific permits (CITES and permits related to national legislation in the countries of origin). Blood and tissue samples were taken by a vet and stored in biobanks as standard procedure after sedating animals for other research purposes. No animals were sedated specifically for this study.

A total of 16 museum specimens, collection dates ranging from 1831 to 1967, was added to the dataset. Samples from museum specimens were taken from the maxilloturbinal bone, unless another sample was more readily available. For details on sample storage and processing, see Supplemental Information 2, Supplemental Tables 4 and 5.

For all available samples, analyses were performed on alignments consisting of a region, containing cytochrome B, tRNAThr, tRNAPro and the left domain for the control region (cytB + ctrl reg.) (1454 bp, 202 sequences), the complete mitogenome (16,756 bp, excluding RS-2 and RS-3, 23 sequences) and an alignment including all sequence data, where ambiguous nucleotides were added to create sequences of equal length. Bayesian analyses were performed using MrBayes v.3.1.2^70^,^71^, using a GTR substitution model with gamma-distributed rate variation across sites and a proportion of invariable sites, and a flat Dirichlet distribution for the substitution rate priors and the state frequency priors, as was determined by MrModeltest2 (v.2.3)^72^. The Markov chain Monte Carlo search was continued for 5,000,000 generations, sampling every 100 generations and discarding the first 25% as burnin. ML analyses were done in Garli^73^, using the same setting as used for MrBayes and support of internal nodes was assessed by 100 bootstrap replications in four independent runs. Branches receiving >0.95 PP in Bayesian analysis and/or 70 bootstrap support in ML and MP analysis are considered to be significantly supported. A haplotype network was created for cytB + ctrl reg. using the median-joining algorithm in Network 4.6.1.1 (available from www.fluxus-engineering.com) with equal weighing of all characters. In addition, we calculated haplotype diversity, nucleotide diversity and pairwaise differences within and between the six main haplogroups (i.e. West Africa, Central Africa, North Africa/Asia, North East Africa, East/Southern Africa, and South West Africa) using Arlequin^74^. Bayesian skyline reconstructions were produced, using BEAST v.1.7.5^75^, using estimated TMRCA for modern lions as derived from earlier runs (i.e. 0.245 Ma, stdev. 0.08).

BEAST v.1.7.5^75^ was used to obtain estimated values for the TMRCA to date splits in the lion tree. Five independent runs of 100 million iterations were performed, discarding the first ten percent of each run as burnin, and using the same model as was used for Bayesian analysis and relaxed molecular clock setting. Fossil evidence for the origin of *P*. *leo* (including *P*. *leo spelaea*) was used for calibration and set to 0.55 Ma (stdev. 0.025)^29^,^76^,^77^,^78^,^79^, with a lognormal distribution for the calibration prior. Convergence of the runs was assessed in Tracer. Logcombiner, Treeannotator and Figtree (available from http://tree.bio.ed.ac.uk/software/figtree/) were used to visualize the results.

## Additional Information

**How to cite this article**: Bertola, L. D. *et al.* Phylogeographic Patterns in Africa and High Resolution Delineation of Genetic Clades in the Lion (*Panthera leo*). *Sci. Rep.* **6**, 30807; doi: 10.1038/srep30807 (2016).

## Supplementary Material

Supplementary Information

## Figures and Tables

**Table 1:** Overview of African mammals for which a distinction between West/Central African populations and populations in East/Southern Africa has been described^52^,^80^.

Order	Species (complex)	(sub)Species	Phylogeography references*	Genetic marker
Taxonomic distinction between West/Central Africa and East/Southern Africa				
Primates	Baboon complex (*Papio)*	5 species	Zinner *et al.*^58^	mtDNA
	Green monkey complex (*Chlorocebus*)	6 species	Haus *et al.*^82^	mtDNA
	Senegal galago (*Galago senegalensis*)	4 subspecies	−	−
Hyracoidea	Rock hyrax complex (*Procavia*)	5 species	−	−
Perissodactyla	Black rhino (*Diceros bicornis*)	4 subspecies	Harley *et al.*^83^	msats
	White rhino (*Ceratotherium simum*)	2 subspecies	−	−
Artiodactyla	Giraffe (*Giraffa camelopardalis*)	9 subspecies	Brown *et al.*^9^; Hassanin *et al.*^84^; Bock *et al.*^32^	mtDNA + msats
	African buffalo (*Syncerus caffer*)	3–4 subspecies	Van Hooft *et al.*^85^; Smitz *et al.*^86^	mtDNA + Y chromosomal msats
	Bushbuck (*Tragelaphus scriptus*)	2 groups, numerous subspecies	Moodley & Bruford^10^	mtDNA
	Greater kudu (*Tragelaphus strepsiceros*)	3 subspecies	−	−
	Eland complex (*Tragelaphys debianus/T*. *oryx*)	2 species	−	−
	Bush duiker (*Sylvicapra grimmia*)	8 groups, numerous subspecies	−	−
	Dwarf antelope complex *Neotragus*	3 species	−	−
	Oribi (*Ourebia ourebi*)	7–13 subspecies	−	−
	Reedbuck complex (*Redunca redunca/R*. *arundinum*)	2 species	−	−
	Mountain reedbuck (*Redunca fulvorufula*)	3 subspecies	−	−
	Kob / Puku complex (*Kobus kob/K*. *vardoni*)	2 species	Lorenzen *et al.*^87^	mtDNA + msats
	Lechwe complex (*Kobus leche/K*.*megaceros*)	2 species	−	−
	Waterbuck (*Kobus ellipsiprymnus*)	2 subspecies	Lorenzen *et al.*^36^	mtDNA + msats
	Red-fronted gazelle (*Eudorcas rufifrons*)	5 subspecies	−	−
	Grant’s gazelle complex (*Nanger*)	3 species	−	−
	Topi (*Damaliscus lunatus*)	5–6 subspecies	−	−
	Hartebeest (*Alcelaphus buselaphusi*)	8 subspecies	Arctander *et al.*^88^; Flagstad *et al.*^11^	mtDNA
	Roan antelope (*Hippotragus equinus*)	2 groups, 6 subspecies	Alpers *et al.*^38^; Matthee & Robinson^89^	mtDNA + msats
	Oryx complex (*Oryx*)	3 species	−	−
Carnivora	Egyptian mongoose (*Herpestes ichneumon*)	up to 11 subspecies	Gaubert *et al.*^90^	mtDNA
	Slender mongoose (*Herpestes sanguineus*)	up to 50 subspecies	−	−
	White-tailed mongoose (*Ichneumia albicauda*)	6 subspecies	Dehghani *et al.*^91^	mtDNA
	Common genet (Genetta genetta)	3 groups, numerous subspecies	Gaubert *et al.*^90^; Delibes & Gaubert (unpub.)	mtDNA
	African civet (*Civettictis civetta*)	5 subspecies	−	−
	Wild cat (*Felis silvestris*)	5 subspecies	Driscoll *et al.*^60^	mtDNA + msats
	Caracal (*Caracal caracal*)	8 subspecies	−	−
	Cheetah (*Acinonyx jubatusi*)	5 infra-specific taxa assessed	Freeman *et al.*^92^; Charruau *et al.*^13^	mtDNA + msats
No taxonomic distinction				
Proboscidea	African (bush) elephant (*Loxodonta africana*)	−	Nyakaana *et al.*^8^	mtDNA + msats
Pholidota	Ground pangolin (*Manis temmicnkii)*	−	−	−
Tubulidentata	Aardvark (*Orycteropus afer*)	−	−	−
Artiodactyla	Common warthog (*Phacochoerus africanus*)	−	Muwanika *et al.*^93^	mtDNA + msats
Carnivora	African wild dog (*Lycaon pictus*)	−	−	−
	Zorilla (*Ictonyx striatus*)	−	−	−
	Honey badger (*Mellivora capensis*)	−	−	−
	Banded mongoose (*Mungos mungo*)	−	−	−
	Marsh mongoose (*Atilax paludinosus*)	−	−	−
	Spotted hyena (*Crocuta crocuta*)	−	Rohland *et al.*^59^; Sheng *et al.*^12^	mtDNA
	Serval (*Leptailurus serval*)	−	−	−
	African Leopard (*Panthera pardus pardus*)	(one subspecies in Africa)	−	−
	African Lion (*Panthera leo leo*)	(one subspecies in Africa)	Dubach *et al.*^17^; Barnett *et al.*^21^; Bertola *et al.*^19^; Bertola *et al.*^24^	mtDNA + msats

For species with phylogenetic data, the used genetic marker is indicated (msats: microsatellites).

^*^Only references that cover the complete (sub)species’s range on the African continent are listed. Publications focusing on a more regional level were excluded.

## References

[b1] Moritz C. & Faith D. P. Comparative phylogeography and the identification of genetically divergent areas for conservation. *Mol*. *Ecol*. 7, 419–429 (1998).

[b2] Bermingham E. & Moritz C. Comparative phylogeography: concepts and applications. *Mol*. *Ecol*. 7, 367–369 (1998).

[b3] Arbogast B. & Kenagy G. J. Comparative phylogeography as an integrative approach to historical biogeography. *J*. *Biogeogr*. 28, 819–825 (2001).

[b4] Hewitt G. M. The structure of biodiversity - insights from molecular phylogeography. *Front*. *Zool*. 1, 4 (2004). 15679920 10.1186/1742-9994-1-4 PMC544936

[b5] Lorenzen E. D., Heller R. & Siegismund H. R. Comparative phylogeography of African savannah ungulates. *Mol*. *Ecol*. 21, 3656–70 (2012). 22702960 10.1111/j.1365-294X.2012.05650.x

[b6] IUCN SSC Cat Specialist Group. Conservation strategy for the lion in West and Central Africa . (IUCN, 2006).

[b7] IUCN SSC Cat Specialist Group. Conservation strategy for the lion (Panthera leo) in Eastern and Southern Africa . (IUCN, 2006).

[b8] Nyakaana S., Arctander P. & Siegismund H. R. Population structure of the African savannah elephant inferred from mitochondrial control region sequences and nuclear microsatellite loci. *Heredity* (*Edinb*) 89, 90–8 (2002). 12136410 10.1038/sj.hdy.6800110

[b9] Brown D. M. *et al.* Extensive population genetic structure in the giraffe. *BMC Biol*. 5, 57 (2007). 18154651 10.1186/1741-7007-5-57 PMC2254591

[b10] Moodley Y. & Bruford M. W. Molecular biogeography: towards an integrated framework for conserving pan-African biodiversity. Plos One 2, e454 (2007). 17520013 10.1371/journal.pone.0000454 PMC1866246

[b11] Flagstad O., Syvertsen P. O., Stenseth N. C. & Jakobsen K. S. Environmental change and rates of evolution: the phylogeographic pattern within the hartebeest complex as related to climatic variation. *Proc*. *Biol*. *Sci*. 268, 667–77 (2001). 11321054 10.1098/rspb.2000.1416 PMC1088655

[b12] Sheng G.-L. *et al.* Pleistocene Chinese cave hyenas and the recent Eurasian history of the spotted hyena, Crocuta crocuta. *Mol*. *Ecol*. 23, 522–33 (2014). 24320717 10.1111/mec.12576

[b13] Charruau P. *et al.* Phylogeography, genetic structure and population divergence time of cheetahs in Africa and Asia: evidence for long-term geographic isolates. *Mol*. *Ecol*. 15, 367–371 (2011). 10.1111/j.1365-294X.2010.04986.x PMC3531615 21214655

[b14] Barnett R., Yamaguchi N., Barnes I. & Cooper A. The origin, current diversity and future conservation of the modern lion (Panthera leo). *Proc*. *R*. *Soc*. *B Biol*. *Sci*. 273, 2119–2125 (2006). 10.1098/rspb.2006.3555 PMC1635511 16901830

[b15] Barnett R., Yamaguchi N., Barnes I. & Cooper A. Lost populations and preserving genetic diversity in the lion Panthera leo: Implications for its *ex situ* conservation. *Conserv*. *Genet*. 7, 507–514 (2006).

[b16] Dubach J. *et al.* Molecular genetic variation across the southern and eastern geographic ranges of the African lion, Panthera leo. *Conserv*. *Genet*. 6, 15–24 (2005).

[b17] Dubach J. M., Briggs M. B., White P. A., Ament B. A. & Patterson B. D. Genetic perspectives on ‘Lion Conservation Units’ in Eastern and Southern Africa. *Conserv*. *Genet*. 1942 (2013).

[b18] Antunes A. *et al.* The evolutionary dynamics of the lion Panthera leo revealed by host and viral population genomics. *Plos Genet*. 4, e1000251 (2008). 18989457 10.1371/journal.pgen.1000251 PMC2572142

[b19] Bertola L. D. *et al.* Genetic diversity, evolutionary history and implications for conservation of the lion (Panthera leo) in West and Central Africa. *J*. *Biogeogr*. 38, 1356–1367 (2011).

[b20] Bruche S. *et al.* A genetically distinct lion (Panthera leo) population from Ethiopia. *Eur*. *J*. *Wildl*. *Res*. 59, 215–225 (2012).

[b21] Barnett R. *et al.* Revealing the maternal demographic history of Panthera leo using ancient DNA and a spatially explicit genealogical analysis. *BMC Evol*. *Biol*. 14, 70 (2014). 24690312 10.1186/1471-2148-14-70 PMC3997813

[b22] Toews D. P. L. & Brelsford A. The biogeography of mitochondrial and nuclear discordance in animals. *Mol*. *Ecol*. 21, 3907–30 (2012). 22738314 10.1111/j.1365-294X.2012.05664.x

[b23] Zink R. M. & Barrowclough G. F. Mitochondrial DNA under siege in avian phylogeography. *Mol*. *Ecol*. 17, 2107–2121 (2008). 18397219 10.1111/j.1365-294X.2008.03737.x

[b24] Bertola L. D. *et al.* Autosomal and mtDNA Markers Affirm the Distinctiveness of Lions in West and Central Africa. Plos One 10, e0137975 (2015). 26466139 10.1371/journal.pone.0137975 PMC4605676

[b25] Henschel P. *et al.* The Lion in West Africa Is Critically Endangered. Plos One 9, e83500 (2014). 24421889 10.1371/journal.pone.0083500 PMC3885426

[b26] Riggio J. *et al.* The size of savannah Africa: a lion’s (Panthera leo) view. *Biodivers*. *Conserv*. 22, 17–35 (2012).

[b27] Vrba E. S. In *Paleoclimate Evol*. *with Emphas*. *Hum*. *Orig*. (Vrba E. S., Denton G. H., Partridge T. C. & Burckle L. H. ) 385–424 (Yale University Press, 1995).

[b28] Barnett R. *et al.* Phylogeography of lions (Panthera leo ssp.) reveals three distinct taxa and a late Pleistocene reduction in genetic diversity. *Mol*. *Ecol*. 18, 1668–1677 (2009). 19302360 10.1111/j.1365-294X.2009.04134.x

[b29] Burger J. *et al.* Molecular phylogeny of the extinct cave lion Panthera leo spelaea. *Mol*. *Phylogenet*. *Evol*. 30, 841–849 (2004). 15012963 10.1016/j.ympev.2003.07.020

[b30] Miller S. M. *et al.* Management of reintroduced lions in small. fenced reserves in South Africa: an assessment and guidelines. *South African J*. *Wildl*. *Res*. 43, 138–154 (2013).

[b31] Levinsky I. *et al.* Climate envelope models suggest spatio-temporal co-occurrence of refugia of African birds and mammals. *Glob*. *Ecol*. *Biogeogr* . 22, 351–363 (2013).

[b32] Bock F. *et al.* Mitochondrial sequences reveal a clear separation between Angolan and South African giraffe along a cryptic rift valley. *BMC Evol*. *Biol*. 14, 219 (2014). 25927851 10.1186/s12862-014-0219-7 PMC4207324

[b33] Moodley Y. & Harley E. H. Population structuring in mountain zebras (Equus zebra): The molecular consequences of divergent demographic histories. *Conserv*. *Genet*. 6, 953–968 (2006).

[b34] Nersting L. G. & Arctander P. Phylogeography and conservation of impala and greater kudu. *Mol*. *Ecol*. 10, 711–9 (2001). 11298982 10.1046/j.1365-294x.2001.01205.x

[b35] Pitra C., Hansen A. J., Lieckefeldt D. & Arctander P. An exceptional case of historical outbreeding in African sable antelope populations. *Mol*. *Ecol*. 11, 1197–1208 (2002). 12074727 10.1046/j.1365-294x.2002.01516.x

[b36] Lorenzen E. D., Simonsen B. T., Kat P. W., Arctander P. & Siegismund H. R. Hybridization between subspecies of waterbuck (Kobus ellipsiprymnus) in zones of overlap with limited introgression. *Mol*. *Ecol*. 15, 3787–99 (2006). 17032274 10.1111/j.1365-294X.2006.03059.x

[b37] Masembe C., Muwanika V. B., Nyakaana S., Arctander P. & Siegismund H. R. Three genetically divergent lineages of the Oryx in eastern Africa: Evidence for an ancient introgressive hybridization. *Conserv*. *Genet*. 7, 551–562 (2006).

[b38] Alpers D. L., Van Vuuren B. J., Arctander P. & Robinson T. J. Population genetics of the roan antelope (Hippotragus equinus) with suggestions for conservation. *Mol*. *Ecol*. 13, 1771–84 (2004). 15189202 10.1111/j.1365-294X.2004.02204.x

[b39] Blome M. W., Cohen A. S., Tryon C. A., Brooks A. S. & Russell J. The environmental context for the origins of modern human diversity: A synthesis of regional variability in African climate 150,000–30,000 years ago. *J*. *Hum*. *Evol*. 1–30, doi: 10.1016/j.jhevol.2012.01.011 (2012). 22513381

[b40] Castañeda I. S. *et al.* Wet phases in the Sahara/Sahel region and human migration patterns in North Africa. *Proc*. *Natl*. *Acad*. *Sci*. *USA* 106, 20159–63 (2009). 19910531 10.1073/pnas.0905771106 PMC2776605

[b41] Gonder M. K., Mortensen H. M., Reed F. A., de Sousa A. & Tishkoff S. A. Whole-mtDNA genome sequence analysis of ancient African lineages. *Mol*. *Biol*. *Evol*. 24, 757–68 (2007). 17194802 10.1093/molbev/msl209

[b42] Tishkoff S. A. *et al.* The genetic structure and history of Africans and African Americans. *Science*. 324, 1035–44 (2009). 19407144 10.1126/science.1172257 PMC2947357

[b43] Templeton A. Out of Africa again and again. Nature 416, 45–51 (2002). 11882887 10.1038/416045a

[b44] Carto S. L., Weaver A. J., Hetherington R., Lam Y. & Wiebe E. C. Out of Africa and into an ice age: on the role of global climate change in the late Pleistocene migration of early modern humans out of Africa. *J*. *Hum*. *Evol*. 56, 139–51 (2009). 19019409 10.1016/j.jhevol.2008.09.004

[b45] Cronin. Paleoclimates. Understanding climate change past and present (Columbia University Press, 2010).

[b46] Dauby G. *et al.* Congruent phylogeographical patterns of eight tree species in Atlantic Central Africa provide insights into the past dynamics of forest cover. *Mol*. *Ecol*. 23, 2299–312 (2014). 24655106 10.1111/mec.12724

[b47] Migliore J. *et al.* Surviving in mountain climate refugia: new insights from the genetic diversity and structure of the relict shrub Myrtus nivellei (Myrtaceae) in the Sahara Desert. Plos One 8, e73795 (2013). 24058489 10.1371/journal.pone.0073795 PMC3776782

[b48] Yamaguchi N., Cooper A., Werdelin L. & Macdonald D. W. Evolution of the mane and group-living in the lion (Panthera leo): a review. *J*. *Zool*. 263, 329–342 (2004).

[b49] Nowell K. & Jackson P. Wild Cats . (IUCN, 1996).

[b50] Clement A. C., Hall A. & Broccoli A. J. The importance of precessional signals in the tropical climate. *Clim*. *Dyn*. 22, 327–341 (2004).

[b51] Imbrie J. *et al.* In *Milankovitch Clim*. *Part 1* (Berger A., Imbrie J., Hays H., Kukla G. & Saltzman B. ) 269–305 (Reidel Publishing, 1984).

[b52] Kingdon J. The Kingdon Field Guide to African Mammals . (A&C Black Publishers, Ltd., 2007).

[b53] Lehmann C. E. R., Archibald S. A., Hoffmann W. A. & Bond W. J. Deciphering the distribution of the savanna biome. *New Phytol*. 191, 197–209 (2011). 21463328 10.1111/j.1469-8137.2011.03689.x

[b54] Staver A. C., Archibald S. & Levin S. Tree cover in sub-Saharan Africa: rainfall and fire constrain forest and savanna as alternative stable states. Ecology 92, 1063–72 (2011). 21661567 10.1890/10-1684.1

[b55] De Vivo M. & Carmignotto A. P. Holocene vegetation change and the mammal faunas of South America and Africa. *J*. *Biogeogr*. 31, 943–957 (2004).

[b56] Hardy O. J. *et al.* Comparative phylogeography of African rain forest trees: A review of genetic signatures of vegetation history in the Guineo-Congolian region. Comptes Rendus Geosci . 345, 284–296 (2013).

[b57] Dupont L. M. & Hooghiemstra H. The Saharan-Sahelian boundary during the Brunhes chron. *Acta Bot*. *Neerl*. 38, 405–415 (1989).

[b58] Zinner D., Groeneveld L. F., Keller C. & Roos C. Mitochondrial phylogeography of baboons (Papio spp.): indication for introgressive hybridization? *BMC Evol*. *Biol*. 9, 83 (2009). 19389236 10.1186/1471-2148-9-83 PMC2681462

[b59] Rohland N. *et al.* The population history of extant and extinct hyenas. *Mol*. *Biol*. *Evol*. 22, 2435–43 (2005). 16120805 10.1093/molbev/msi244

[b60] Driscoll C. A. *et al.* The Near Eastern origin of cat domestication. Science 317, 519–23 (2007). 17600185 10.1126/science.1139518 PMC5612713

[b61] Dupont L. Orbital scale vegetation change in Africa. *Quat*. *Sci*. *Rev*. 30, 3589–3602 (2011).

[b62] Petit J. R. *et al.* Climate and atmospheric history of the past 420,000 years from the Vostok ice core. Antarctica. Nature 399, 429–436 (1999).

[b63] Van Andel T. H. & Tzedakis P. C. Palaeolithic landscapes of Europe and environs, 150,000-25,000 years ago: an overview. *Quat*. *Sci*. *Rev*. 15, 481–500 (1996).

[b64] Hooghiemstra H., Agwu C. O. C. & Dupont L. M. Vegetational and climatic changes at the northern fringe of the Sahara 250,000-5000 years BP: evidence from 4 marine pollen records located between Portugal and the Canary Islands. *Rev*. *Paleobotany Palynol* . 74, 1–53 (1992).

[b65] Hoelzmann P. *et al.* In *Past Clim*. *Var*. *through Eur*. *Africa* (Battarbee R. W., Gasse F. & Stickley C. E. ) 219–256 (Springer, 2004).

[b66] Thapar V., Thapar R. & Ansari Y. Exotic Aliens: The lion & the cheetah in India . (Aleph Book Company, 2013).

[b67] Mazák J. H. Geographical variation and phylogenetics of modern lions based on craniometric data. *J*. *Zool*. 281, 194–209 (2010).

[b68] Hemmer H. Untersuchungen zur Stammesgeschichte der Pantherkatzen (Pantherinae) 3 Zur Artgeschichte des Löwen Panthera (Panthera) leo (Linnaeus 1758) . (Veröffentlichungen der Zoologischen Staatssammlung München, 1974).

[b69] Moritz C. Defining ‘Evolutionarily Significant Units’ for conservation. *Trends Ecol*. *Evol*. 9, 373–375 (1994). 21236896 10.1016/0169-5347(94)90057-4

[b70] Huelsenbeck J. P. & Ronquist F. MRBAYES: Bayesian inference of phylogenetic trees. Bioinformatics 17, 754–5 (2001). 11524383 10.1093/bioinformatics/17.8.754

[b71] Ronquist F. *et al.* MrBayes 3.2: efficient Bayesian phylogenetic inference and model choice across a large model space. *Syst*. *Biol*. 61, 539–42 (2012). 22357727 10.1093/sysbio/sys029 PMC3329765

[b72] Nylander J. A. A. MrModeltest v2.3. Program distributed by the author (https://github.com/nylander/MrModeltest2). Evolutionary Biology Centre, Uppsala University, Uppsala, Sweden (2004).

[b73] Zwickl D. J. Genetic algorithm approaches for the phylogenetic analysis of large biological sequence datasets under the maximum likelihood criterion. PhD thesis, University of Texas at Austin (2006).

[b74] Excoffier L., Laval G. & Schneider S. Arlequin (version 3.0): an integrated software package for population genetics data analysis. *Evol*. *Bioinform*. *Online* 1, 47–50 (2005). 19325852 PMC2658868

[b75] Drummond A. J. & Rambaut A. BEAST: Bayesian evolutionary analysis by sampling trees. *BMC Evol*. *Biol*. 7, 214 (2007). 17996036 10.1186/1471-2148-7-214 PMC2247476

[b76] Johnson W. E. *et al.* The late Miocene radiation of modern Felidae: a genetic assessment. *Science* (*80-*) 311, 73–77 (2006). 10.1126/science.1122277 16400146

[b77] Janczewski D. N., Modi W. S., Stephens J. C. & O’Brien S. J. Mitochondrial 12S molecular evolution of mitochondrial 12S RNA and and cytochrome b sequences in pantherine lineage of Felidae. *Mol*. *Biol*. *Evol*. 12, 690–707 (1995). 7544865 10.1093/oxfordjournals.molbev.a040232

[b78] Davis B. W., Li G. & Murphy W. J. Supermatrix and species tree methods resolve phylogenetic relationships within the big cats, Panthera (Carnivora: Felidae). *Mol*. *Phylogenet*. *Evol*. 56, 64–76 (2010). 20138224 10.1016/j.ympev.2010.01.036

[b79] Kurten B. & Anderson E. Pleistocene mammals of North America . (Columbia University Press, 1980).

[b80] IUCN. The IUCN Red List of Threatened Species. Version 2014.1. at http://www.iucnredlist.org (2014).

[b81] Lisiecki L. E. & Raymo M. E. A Pliocene-Pleistocene stack of 57 globally distributed benthic δ 18 O records. Paleoceanography 20, (2005).

[b82] Haus T. *et al.* Mitochondrial diversity and distribution of african green monkeys (chlorocebus gray, 1870). *Am*. *J*. *Primatol*. 75, 350–60 (2013). 23307319 10.1002/ajp.22113 PMC3613741

[b83] Harley E. H., Baumgarten I., Cunningham J. & O’Ryan C. Genetic variation and population structure in remnant populations of black rhinoceros, Diceros bicornis, in Africa. *Mol*. *Ecol*. 14, 2981–90 (2005). 16101768 10.1111/j.1365-294X.2005.02660.x

[b84] Hassanin A., Ropiquet A., Gourmand A.-L., Chardonnet B. & Rigoulet J. Mitochondrial DNA variability in Giraffa camelopardalis: consequences for taxonomy, phylogeography and conservation of giraffes in West and central Africa. *C*. *R*. *Biol*. 330, 265–74 (2007). 17434121 10.1016/j.crvi.2007.02.008

[b85] Van Hooft W. F., Groen A. F. & Prins H. H. T. Phylogeography of the African buffalo based on mitochondrial and Y-chromosomal loci: Pleistocene origin and population expansion of the Cape buffalo subspecies. *Mol*. *Ecol*. 11, 267–79 (2002). 11856427 10.1046/j.1365-294x.2002.01429.x

[b86] Smitz N. *et al.* Pan-African Genetic Structure in the African Buffalo (Syncerus caffer): Investigating Intraspecific Divergence. Plos One 8, e56235 (2013). 23437100 10.1371/journal.pone.0056235 PMC3578844

[b87] Lorenzen E. D., De Neergaard R., Arctander P. & Siegismund H. R. Phylogeography, hybridization and Pleistocene refugia of the kob antelope (Kobus kob). *Mol*. *Ecol*. 16, 3241–52 (2007). 17651200 10.1111/j.1365-294X.2007.03382.x

[b88] Arctander P., Johansen C. & Coutellec-Vreto M. A. Phylogeography of three closely related African bovids (tribe Alcelaphini). *Mol*. *Biol*. *Evol*. 16, 1724–39 (1999). 10605114 10.1093/oxfordjournals.molbev.a026085

[b89] Matthee C. A. & Robinson T. J. Mitochondrial DNA population structure of roan and sable antelope: implications for the translocation and conservation of the species. *Mol*. *Ecol*. 8, 227–38 (1999). 10065541 10.1046/j.1365-294x.1999.00556.x

[b90] Gaubert P. *et al.* Comparative phylogeography of two African carnivorans presumably introduced into Europe: disentangling natural versus human-mediated dispersal across the Strait of Gibraltar. *J*. *Biogeogr*. 38, 341–358 (2011).

[b91] Dehghani R. *et al.* Phylogeography of the white-tailed mongoose (Herpestidae, Carnivora, Mammalia) based on partial sequences of the mtDNA control region. *J*. *Zool*. 276, 385–393 (2008).

[b92] Freeman A. R. *et al.* Sequence variation in the mitochondrial DNA control region of wild African cheetahs (Acinonyx jubatus). *Heredity* (*Edinb*). 86, 355–62 (2001). 11488972 10.1046/j.1365-2540.2001.00840.x

[b93] Muwanika V. B., Nyakaana S., Siegismund H. R. & Arctander P. Phylogeography and population structure of the common warthog (Phacochoerus africanus) inferred from variation in mitochondrial DNA sequences and microsatellite loci. *Heredity* (*Edinb*). 91, 361–72 (2003). 14512951 10.1038/sj.hdy.6800341

