# Supplementary material for: Phylogeographic Patterns in Africa and High Resolution Delineation of Genetic Clades in the Lion (Panthera leo)
Source: Sci Rep. 2016 Aug 4;6:30807. doi: 10.1038/srep30807 (PMC4973251; doi:10.1038/srep30807)
Supplement: Supplementary Information [file srep30807-s1.pdf]

Supporting Information

Phylogeographic Patterns in Africa and High Resolution Delineation of Genetic Clades in the Lion (*Panthera leo*)

L. D. Bertola[1,2*], H. Jongbloed[1,2], K.J. van der Gaag[3], P. de Knijff[3], N. Yamaguchi[4], H. Hooghiemstra[5], H. Bauer[6], P. Henschel[7], P.A. White[8], C.A. Driscoll[9], T.Tende[10], U. Ottosson[10], Y. Saidu[11], K. Vrieling[2,¶] and H. H. de Iongh[1,12,¶]

[1] *Leiden University, Institute of Environmental Sciences (CML), PO Box 9518, 2300 RA Leiden, The Netherlands*

[2] *Leiden University, Institute of Biology Leiden (IBL), PO Box 9505, 2300 RA Leiden, The Netherlands*

[3] *Forensic Laboratory for DNA Research, Department of Human Genetics, Leiden University Medical Centre, P.O. Box 9600, 2300 RC Leiden, The Netherlands*

[4] *Qatar University, Department of Biological and Environmental Sciences, College of Arts and Sciences, PO Box 2713, Doha, Qatar*

[5] *Institute for Biodiversity and Ecosystem Dynamics, University of Amsterdam, Science Park 904, 1018 XH Amsterdam, The Netherlands*

[6] *WildCRU, Recanati-Kaplan Centre, University of Oxford. Tubney House, Abingdon Road, OX13 5QL, UK*

[7] *Panthera, 8 West 40th Street, 18th Floor, New York, NY 10018, USA*

[8] *Center for Tropical Research, Institute of the Environment and Sustainability, University of California, Los Angeles, CA 90095-1496, USA*

[9] *Wildlife Institute of India, Dehradun 248 001, Uttarakhand, India*

[10] *A. P. Leventis Ornithological Research Institute, P.O. Box 13404 Jos, Nigeria*

[11] *Nigeria National Park Service, PMB 0258 Garki-Abuja, Nigeria*

[12] *University of Antwerp, Department Biology, Evolutionary Ecology Group, Groenenborgerlaan 171, 2020 Antwerpen, Belgium*

Supplemental Table 1. Lion samples and outgroup sequences used in this study.

Number	Country	Location	Region	Source	Literature
1	Senegal	Niokolo-Koba NP	West	Wild	Bertola et al., 2015
2	Senegal	Niokolo-Koba NP	West	Wild	Bertola et al., 2015
3	Senegal	Niokolo-Koba NP	West	Wild	Bertola et al., 2015
4	Senegal	Niokolo-Koba NP	West	Wild	Bertola et al., 2015
5	Senegal	Niokolo-Koba NP	West	Wild	Bertola et al., 2015
6	Senegal	Niokolo-Koba NP	West	Wild	Bertola et al., 2015
7	Senegal	Niokolo-Koba NP	West	Wild	Bertola et al., 2015
8	Guinea	-	West	Wild	this study
9	Benin	Pendjari NP	West	Wild	this study
10	Benin	Pendjari NP	West	Wild	Bertola et al., 2011
11	Benin	Pendjari NP	West	Wild	Bertola et al., 2015
12	Benin	Pendjari NP	West	Wild	Bertola et al., 2015
13	Benin	Pendjari NP	West	Wild	Bertola et al., 2015
14	Nigeria	Yankari GR	West	Wild	this study
15	Nigeria	Yankari GR	West	Wild	this study
16	Nigeria	Yankari GR	West	Wild	this study
17	Nigeria	Yankari GR	West	Wild	this study
18	Nigeria	Kainji NP	West	Wild	this study
19	Nigeria	Kainji NP	West	Wild	this study
20	Cameroon	Waza NP	Central	Wild	Bertola et al., 2011
21	Cameroon	Waza NP	Central	Wild	this study
22	Cameroon	Waza NP	Central	Wild	Bertola et al., 2011
23	Cameroon	Waza NP	Central	Wild	Bertola et al., 2011
24	Cameroon	Waza NP	Central	Wild	Bertola et al., 2011
25	Cameroon	Waza NP	Central	Wild	Bertola et al., 2011
26	Cameroon	Waza NP	Central	Wild	Bertola et al., 2011
27	Cameroon	Waza NP	Central	Wild	Bertola et al., 2011
28	Cameroon	Waza NP	Central	Wild	Bertola et al., 2011
29	Cameroon	Faro NP	Central	Wild	this study
30	Cameroon	Faro NP	Central	Wild	this study
31	Cameroon	Bénoué NP	Central	Wild	this study
32	Cameroon	Bénoué NP	Central	Wild	Bertola et al., 2011
33	Cameroon	Bouba Njida NP	Central	Wild	this study
34	Cameroon	Bouba Njida NP	Central	Wild	this study
35	Cameroon	Bouba Njida NP	Central	Wild	this study
36	Chad	Zakouma NP	Central	Wild	Bertola et al., 2011
37	Chad	Zakouma NP	Central	Wild	Bertola et al., 2011
38	Chad	Zakouma NP	Central	Wild	Bertola et al., 2011
39	Chad	Zakouma NP	Central	Wild	Bertola et al., 2011
40	CAR	Birao	Central	Museum (I)/Wild	this study
41	DRC	Garamba NP	Central	Wild	Bertola et al., 2015
42	DRC	Garamba NP	Central	Wild	this study
43	DRC	Garamba NP	Central	Wild	Bertola et al., 2015
44	DRC	Garamba NP	Central	Wild	Bertola et al., 2015
45	DRC	Garamba NP	Central	Wild	Bertola et al., 2015
46	DRC	Garamba NP	Central	Wild	Bertola et al., 2015
47	DRC	-	Central	Captive (i)	this study
48	DRC	-	Central	Captive (i)	Bertola et al., 2011
49	DRC	Ruindi Plains S. of Lake Edward	Central	Museum (II)/Wild	this study
50	Ethiopia	Gambela NP	East	Wild	this study
51	Ethiopia	Kaffa Province	East	Wild	this study
52	Ethiopia	Nechisar NP	East	Wild	this study
53	Ethiopia	Nechisar NP	East	Wild	this study
54	Ethiopia	Bale Mountains NP	East	Wild	this study
55	Ethiopia	Oromia region, Hudet	East	Wild	this study
56	Ethiopia	Somali region, Dolo Ado	East	Wild	this study
57	Ethiopia	Somali region, Kebri Dehar	East	Wild	this study
58	Ethiopia	Somali region	East	Wild/Captive (ii)	this study
59	Ethiopia	Somali region	East	Wild/Captive (ii)	this study
60	Ethiopia	-	East	Captive (iii)	this study
61	Ethiopia	-	East	Captive (iii)	Bertola et al., 2015
62	Ethiopia	-	East	Captive (iii)	Bertola et al., 2015
63	Ethiopia	-	East	Captive (iii)	Bertola et al., 2015
64	Ethiopia	-	East	Captive (iii)	Bertola et al., 2015
65	Ethiopia	-	East	Captive (iv)	Bertola et al., 2011
66	Ethiopia	-	East	Captive (iv)	Bertola et al., 2011
67	Ethiopia	-	East	Captive (iv)	Bertola et al., 2011
68	Ethiopia	-	East	Captive (iv)	Bertola et al., 2011
69	Somalia	-	East	Captive (v)	Bertola et al., 2011
70	Somalia	-	East	Captive (v)	Bertola et al., 2011
71	Somalia	-	East	Captive (vi)	this study
72	Kenya	Aberdare NP	East	Wild	this study
73	Kenya	Maasai Mara NR	East	Wild	this study
74	Kenya	Maasai Mara NR	East	Wild	this study
75	Kenya	Maasai Mara NR	East	Wild	this study
76	Kenya	Maasai Mara NR	East	Wild	this study
77	Kenya	Maasai Mara NR	East	Wild	this study

78	Kenya	Maasai Mara NR	East	Wild	this study
79	Kenya	Amboseli NP	East	Wild	Bertola et al., 2015
80	Kenya	Amboseli NP	East	Wild	Bertola et al., 2015
81	Kenya	Amboseli NP	East	Wild	Bertola et al., 2015
82	Kenya	Amboseli NP	East	Wild	Bertola et al., 2015
83	Kenya	Amboseli NP	East	Wild	Bertola et al., 2015
84	Kenya	Amboseli NP	East	Wild	Bertola et al., 2015
85	Kenya	Amboseli NP	East	Wild	Bertola et al., 2015
86	Kenya	Kuku group ranch	East	Wild	this study
87	Kenya	Tsavo East NP	East	Wild	this study
88	Kenya	Tsavo East NP	East	Wild	this study
89	Kenya	Tsavo East NP	East	Wild	this study
90	Kenya	Tsavo East NP	East	Wild	this study
91	Tanzania	Serengeti NP	East	Wild	Bertola et al., 2015
92	Tanzania	Serengeti NP	East	Wild	Bertola et al., 2015
93	Tanzania	Serengeti NP	East	Wild	Bertola et al., 2015
94	Tanzania	Ngorongoro Conservation Area	East	Wild	Bertola et al., 2015
95	Zambia	Mpika town	South	Wild	this study
96	Zambia	Mulobezi town	South	Wild	this study
97	Zambia	Mumbwa town	South	Wild	this study
98	Zambia	north of Lusaka	South	Wild	this study
99	Zambia	Luangwa valley	South	Wild	Bertola et al., 2015
100	Zambia	Luangwa valley	South	Wild	Bertola et al., 2015
101	Zambia	Luangwa valley	South	Wild	Bertola et al., 2015
102	Zambia	Luangwa valley	South	Wild	Bertola et al., 2015
103	Zambia	Luangwa valley	South	Wild	Bertola et al., 2015
104	Zambia	Luangwa valley	South	Wild	Bertola et al., 2015
105	Zambia	Luangwa valley	South	Wild	Bertola et al., 2015
106	Zambia	Luangwa valley	South	Wild	Bertola et al., 2015
107	Zambia	Luangwa valley	South	Wild	Bertola et al., 2015
108	Zambia	Luangwa valley	South	Wild	this study
109	Zambia	Luangwa valley	South	Wild	this study
110	Zambia	Luangwa valley	South	Wild	this study
111	Zambia	Victoria Falls	South	Museum (III)/Wild	this study
112	Zambia	Mweru Wantipa	South	Museum (IV)/Wild	this study
113	Zimbabwe	Save Valley Conservancy	South	Wild	this study
114	Zimbabwe	Save Valley Conservancy	South	Wild	this study
115	Zimbabwe	Save Valley Conservancy	South	Wild	this study
116	Zimbabwe	Save Valley Conservancy	South	Wild	this study
117	Zimbabwe	Nuanetsi area	South	Museum (V)/Wild	this study
118	Zimbabwe	Robin's Camp	South	Museum (V)/Wild	this study
119	Botswana	-	South	Captive (vii)	Bertola et al., 2011
120	Botswana	-	South	Captive (vii)	this study
121	Botswana	-	South	Captive (vii)	Bertola et al., 2011
122	Botswana	Aha Hills	South	Museum (III)/Wild	this study
123	Mozambique	Caia	South	Museum (VI)/Wild	this study
124	Mozambique	Changara	South	Museum (III)/Wild	this study
125	Angola	-	South	Museum (VI)/Wild	this study
126	Namibia	Etosha NP	South	Wild	Bertola et al., 2015
127	Namibia	Etosha NP	South	Wild	Bertola et al., 2015
128	Namibia	Easter Caprivi	South	Wild	this study
129	Namibia	East Etosha	South	Wild	this study
130	Namibia	East and West Caprivi/Botswana	South	Wild	this study
131	Namibia	Eastern Etosha	South	Wild	this study
132	Namibia	Eastern Etosha	South	Wild	this study
133	Namibia	West Caprivi and Angola	South	Wild	this study
134	Namibia	Etosha Central	South	Wild	this study
135	Namibia	East Etosha	South	Wild	this study
136	Namibia	Erongo/Walvis Bay	South	Museum (II)/Wild	this study
137	Namibia	-	South	Captive (viii)	this study
138	Namibia	-	South	Captive (viii)	Bertola et al., 2011
139	RSA	Kalahari Gemsbok NP	South	Wild	Bertola et al., 2015
140	RSA	Kalahari Gemsbok NP	South	Wild	Bertola et al., 2015
141	RSA	Kgalagadi to Tswalu	South	Wild	this study
142	RSA	Kgalagadi to Tswalu	South	Wild	this study
143	RSA	Kgalagadi Transfrontier Park	South	Wild	this study
144	RSA	Kgalagadi Transfrontier Park	South	Wild	this study
145	RSA	Kruger NP: Gogonthaba, Malelane	South	Wild	Bertola et al., 2015
146	RSA	Kruger NP: Gogonthaba, Malelane	South	Wild	Bertola et al., 2015
147	RSA	Kruger NP: Skukuza Phabeni/ Nwaswitshaka watergat pad junction	South	Wild	Bertola et al., 2015
148	RSA	Kruger NP: Skukuza Phabeni/ Nwaswitshaka watergat pad junction	South	Wild	Bertola et al., 2015
149	RSA	Kruger NP: Pretoriuskop, Fayi loop	South	Wild	Bertola et al., 2015
150	RSA	Kruger NP: Lower Sabie, S128	South	Wild	Bertola et al., 2015

151	RSA	Kruger NP: Crocodile Bridge	South	Wild	Bertola et al., 2015
152	RSA	Kruger NP: Crocodile Bridge	South	Wild	Bertola et al., 2015
153	RSA	Kruger NP: Stolznek, North of Biyamiti	South	Wild	Bertola et al., 2015
154	RSA	Kruger NP: Stolznek	South	Wild	Bertola et al., 2015
155	RSA	Venetia-Limpopo NR, Tuli Block	South	Wild	this study
156	RSA	Venetia-Limpopo NR, Tuli Block	South	Wild	this study
157	RSA	Venetia-Limpopo NR, Tuli Block	South	Wild	this study
158	RSA	Venetia-Limpopo NR, Tuli Block	South	Wild	this study
159	RSA	Venetia-Limpopo NR, Tuli Block	South	Wild	this study
160	RSA	Venetia-Limpopo NR, Tuli Block	South	Wild	this study
161	RSA	Kruger NP: Timbavati	South	Captive (ix)	Bertola et al., 2011
162	RSA	Kruger NP: Timbavati	South	Captive (ix)	this study
163	RSA	Kruger NP: Timbavati	South	Captive (ix)	Bertola et al., 2011
164*	RSA	-	South	Museum (I)/Wild	this study
165	Barbary	-	North	Museum (II)/Wild	this study
166	Barbary	-	North	Museum (VI)/Wild	this study
167*	Middle-East	-	North	Museum (VII)/Captive	this study
168*	Middle-East	-	North	Museum (VII)/Captive	this study
169	India	Gir Forest NP	India	Wild	Bertola et al., 2015
170	India	Gir Forest NP	India	Wild	Bertola et al., 2015
171	India	Gir Forest NP	India	Wild	Bertola et al., 2015
172	India	Gir Forest NP	India	Wild (Captive born (x), founders both Wild)	Bertola et al., 2015
173	India	Gir Forest NP	India	Wild (Captive born (x), founders both Wild)	Bertola et al., 2015
174	India	Gir Forest NP	India	Wild (Captive born (x), founders both Wild)	this study
175	India	Gir Forest NP	India	Captive (i)	Bertola et al., 2011
176	India	Gir Forest NP	India	Captive (i)	Bertola et al., 2011
177	India	Gir Forest NP	India	Captive (i)	Bertola et al., 2011
178	India	Gir Forest NP	India	Captive (xi)	Bertola et al., 2011
179	Senegal (PL1)	-	West	GenBank	Barnett et al., 2006+2014
180	Senegal (PL2)	-	West	GenBank	Barnett et al., 2006+2014
181	Barbary (PL3)	-	North	GenBank	Barnett et al., 2006+2014
182	Burkina Faso (PL4)	-	West	GenBank	Barnett et al., 2006+2014
183	Tunisia (PL5)	-	North	GenBank	Barnett et al., 2006+2014
184	North Africa (PL6)	-	North	GenBank	Barnett et al., 2006+2014
185	Algeria (PL7)	-	North	GenBank	Barnett et al., 2006+2014
186	Iran (PL8)	-	North	GenBank	Barnett et al., 2006+2014
187	Iran (PL9)	-	North	GenBank	Barnett et al., 2006+2014
188	North Africa "Tower of London" (PL11)	-	North	GenBank	Barnett et al., 2006+2014
189	North Africa "Tower of London" (PL12)	-	North	GenBank	Barnett et al., 2006+2014
190	Sudan (PL13)	-	Central	GenBank	Barnett et al., 2006+2014
191	CAR (PL15)	-	Central	GenBank	Barnett et al., 2006+2014
192	CAR (PL16)	-	Central	GenBank	Barnett et al., 2006+2014
193	Asiatic lion (*Panthera leo persica*)	India	India	GenBank	Bagatharia et al., 2013
194	Asiatic lion (Panthera leo persica)	India	India	GenBank	Bagatharia et al., 2013
195	Cave lion (*Panthera leo spelaea*)	Germany	Outgroup	GenBank	Burger et al. (2004) + Barnett et al. (2009)
196	Leopard (*Panthera pardus*)	Amur	Outgroup	Captive (xi)	this study
197	Leopard (*Panthera pardus*)	Unknown	Outgroup	GenBank	Wei et al. (2011)
198	Tiger (*Panthera tigris*)	Bengal	Outgroup	GenBank	Kitpipit et al. (2011)
199	Tiger (*Panthera tigris*)	Bengal	Outgroup	GenBank	Kitpipit et al. (2011)
200	Tiger (*Panthera tigris*)	Sumatra	Outgroup	GenBank	Kitpipit et al. (2011)
201	Tiger (*Panthera tigris*)	Sumatra	Outgroup	GenBank	Kitpipit et al. (2011)
202	Tiger (*Panthera tigris*)	Amur	Outgroup	GenBank	Kitpipit et al. (2011)
203	Tiger (*Panthera tigris*)	Amur	Outgroup	GenBank	Kitpipit et al. (2011)
204	Snow leopard (*Panthera uncia*)	Unknown	Outgroup	GenBank	Wei et al. (2011)
205	Clouded leopard (*Neofelis nebulosa*)	Unknown	Outgroup	GenBank	Wu et al. (2007)

* Excluded from analyses presented in the main text. See Supplemental Information 2 for background information and additional analyses.

**SOURCE**	**MUSEUM**	I: Zoological Museum, University of Amsterdam, Amsterdam, The Netherlands / Naturalis Biodiversity Center, Leiden, The Netherlands
		II: Swedish Museum of Natural History, Stockholm, Sweden
		III: Smithsonian: Smithsonian Institution, Washington D.C., U.S.A.
		IV: Brussels: Royal Belgian Institute of Natural Sciences, Brussels, Belgium
		V: Bulawayo: Natural History Museum of Zimbabwe, Bulawayo, Zimbabwe
		VI: Naturalis Biodiversity Center, Leiden, The Netherlands
		VII: Humboldt: Museum für Naturkunde (MfN)/Humboldt Museum, Berlin, Germany
	**CAPTIVE**	i: Diergaarde Blijdorp, Rotterdam, The Netherlands
		ii: BornFree Ethiopia, lions confiscated from the Presidential Palace in Addis Ababa, Ethiopia
		iii: Addis Ababa Lion Zoo, Addis Ababa, Ethiopia
		iv: Sanaa Zoo, Sanaa, Yemen
		v: Safaripark Beekse Bergen, Hilvarenbeek, The Netherlands
		vi: Confiscated individual, Breeding Centre, UAE
		vii: Dierenpark Amersfoort, Amersfoort, The Netherlands
		viii: Zoo Basel, Basel, Switserland
		ix: Ouwehands dierenpark, Rhenen, The Netherlands
		x: Sakkarbaug Zoo: Sakkarbaug Zoological Garden, Junagadh, Gujarat, India
		xi: Planckendael: Planckendael, Muizen, Belgium

Supplemental Figure 1. Phylogenetic tree Mitogenomes.

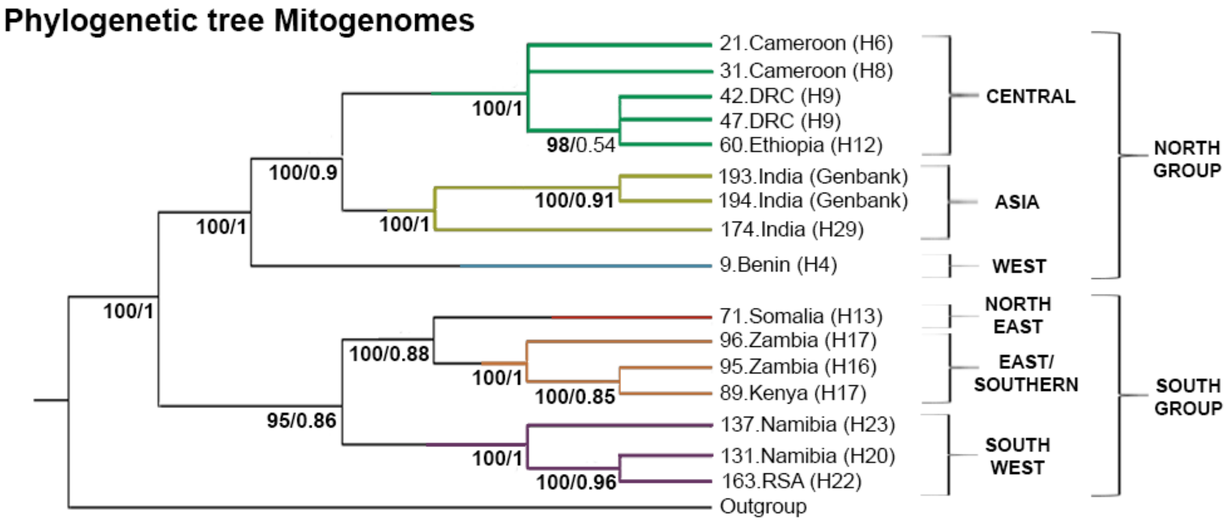

Supplemental Figure 1. Phylogenetic tree of 16 lion populations throughout their complete geographic range, based on their complete mitochondrial genomes. Branch colours correspond to haplotype colours in Figure 3 and 4. Support is indicated as posterior probability (Bayesian analysis)/bootstrap support (ML analysis). Branches are labeled with sample name and corresponding haplotype number (H), as used in Figure 3 and 4.

Supplemental Table 2. Diversity indices for the six main haplogroups based on all cytb+ctrl reg. lion sequences included in this study.
No. samples: number of samples included in the clade; No. haplotypes: number of haplotypes identified within the clade; No. mutations: number of point mutations between haplotypes within the haplogroup; Average number of pairwise differences: between clades, uncorrected (PiXY) (above diagonal), within clades (PiX) (diagonal elements), between clades, corrected (PiXY-(PiX+PiY)/2) (below diagonal)

	No. samples	No. haplotypes	No. mutations	Average number of pairwise differences					
				West	Central	North	North East	East/Souther	South
**West**	18	3	3	1.14379	4.94444	5.44444	12.94444	11.00926	15.06944
**Central**	45	8	8	3.60891	1.52727	7.1	14.14286	12.2	16.26667
**North Africa/Asia**	20	6	8	3.95676	5.42057	1.83158	14.83571	12.9	16.9125
**North East**	14	2	4	11.49343	12.5001	13.0408	1.75824	8.34286	12.51786
**East/Southern**	60	4	4	10.21533	11.21433	11.76218	7.2417	0.44407	10.575
**South West**	32	5	12	12.84231	13.84779	14.34147	9.98349	8.69772	3.31048

Supplemental Table 3. Results of estimates for divergence times for lion clades in years ago (ka), compared to estimates from previous publications. Constraints include the approach and calibration points used. Names of the clades refer to the ingroups.

	Node (Fig. 3)	Age of nodes (95% HPD)			
		This study	Burger et al., 2004	Antunes et al., 2008	Barnett et al., 2014
**Software**		BEAST	r8s	Lintree	BEAST
**Data**		complete mtDNA (total: 17 kbp) + 1.4 kbp fragments	mtDNA (total: 1.1 kbp)	mtDNA (total: 1.9 kbp)	mtDNA (total: 1.1 kbp)
**Constraints**		0.55 (Cave lion - Lion); 1.6 (Leopard-Lion); 3.8 (Panthera) million	clock-based estimate	clock-based estimate	0.55 (Cave lion - Lion) million
**Panthera**	not shown	3.443 (2.590-4.373) million	1.428–2.295 million		
**Leopard - Lion**	not shown	1.469 (1.135-1.812) million	1–1.559 million		
**Cave lions - Lion**	not shown	556.9 ka (510.8-606.1)			
**Lion**	a	291.7 ka (178.0-417.7)	74.0-203.0 ka	324.0 ka (145.0-502.0)	124.2 ka (81.8-183.5)
**South**	b	231.3 ka (132.3-338.7)		split not detected	81.9 ka (45.7-122.2)
**East/Southern - North East**	c	183.7 (107.0-271.1)		split not detected	57.8 ka (26.8-96.6)
**North**	d	174.7 ka (94.9-276.7)		118.0 ka (28.0-208.0)	61.5 ka (32.7-97.3)
**Central - India**	e	141.1 ka (71.7 - 216.6)		split not detected	split not detected
**India - North Africa**	f	110.8 ka (39.7-200.1)		split not detected	21.1 ka (8.3-38.8)
**South West**	g	113.8 ka (55.2-189.0)		169.0 ka (34.0-304.0)	
**East/Southern**	h	78.1 ka (37.2-132.1)		101.0 ka (11.0-191.0)	
**North East**	i	63.9 ka (18.0-118.6)		split not detected	
**West - India**	-	split not detected		split not detected	51.0 ka (26.6-83.1)
**West**	j	63.4 ka (15.1-129.1)		split not detected	
**Central**	k	49.6 ka (20.7-91.0)		split not detected	

Supplemental Information 1. Data authenticity.

Samples from zoos and museums were only included in our study when sufficient information was available on the origin of the individual or its free-ranging ancestors. Our results show that lion populations that were previously described as unique, as was the case for the Addis Ababa lions [1] and for the Sabi Sands lions [2] are most likely the result of incomplete sampling. Angola is represented by one aDNA sample only, which clusters to the South West group. Although it is difficult to draw conclusions for the entire Angolan lion population, this suggests that the captive Angolan lions that were included in previous phylogenetic studies [3–6] are not pure-bred Angolan. Pedigree information also shows that there is no complete documentation of the female lineage in this captive population [7].

In four cases, samples included in our analyses showed unexpected results from the phylogenetic analyses. Since the origin of three samples could not be reconfirmed, they were excluded from analyses presented in the main text. For completeness, results of the analyses including these samples are shown below. In all cases, unique point mutations were double checked by independent PCR and sequencing and laboratory procedures were checked to exclude the possibility of contamination. Addition of these samples does not change the conclusions presented in the main text.

Haplotype 14: Ethiopia captive population (65-68 Ethiopia). This population is located on a long branch, clustering with the North East group. Despite relatively dense sampling of the region, no intermediate haplotypes were identified. Clustering based on mtDNA data and microsatellite data do not contradict the origin of these samples (Bertola et al., submitted). These data were therefore included in all analyses.

Haplotype 23: Namibia captive population (137-138.Namibia). This population is located on a long branch in the South West group, with undetected intermediate haplotypes. Phylogenetic analyses place the population on the expected branch, in the South West group. These data were therefore included in all analyses.

Museum sample 164.RSA (Haplotype *). This sequence was placed in the North East group, with data from Ethiopia, Somalia and Central Kenya. Apart from this specimen, all included samples from the southern part of Kenya and further southward cluster with either East/Southern or the South West group. No samples from the North East group had been processed parallel to this sample and therefore we exclude the possibility of contamination. The specimen was collected by the late L. de Beaufort and comparing this entry to other specimen collected by L. de Beaufort, this entry contained very little information. Because of doubts regarding the authenticity of this entry, and the

unexpected position in the phylogenetic tree, this sample was excluded from the phylogenetic analyses presented in the main text. Results for Bayesian, Maximum Likelihood, Network and BEAST analyses including this sample, are shown below (Supplemental Figures 2-1 and 2-2).

Museum samples 167-168.Middle East (Haplotype 9 and **). These specimen were labeled as hybrids between an Abyssinian male and a female from Mesopotamia (first generation zoo animals). They share a haplotype or cluster close to a haplotype from Central Africa. In contrast, the remaining ten sequences from North Africa and Iran cluster strongly with the Asiatic subspecies. No samples from the Central Africa group had been processed parallel to this sample and therefore we exclude the possibility of contamination. Regarding the sparse information about zoo populations in those times and the unexpected position in the phylogenetic tree, these specimen were excluded from the phylogenetic analyses presented in the main text. Haplotype 9 was retained, since this was found in several other samples, from Central Africa. Results for Bayesian, Maximum Likelihood, Network and BEAST analyses including this sample, are shown below (Supplemental Figures 2-1 and 2-2).

a

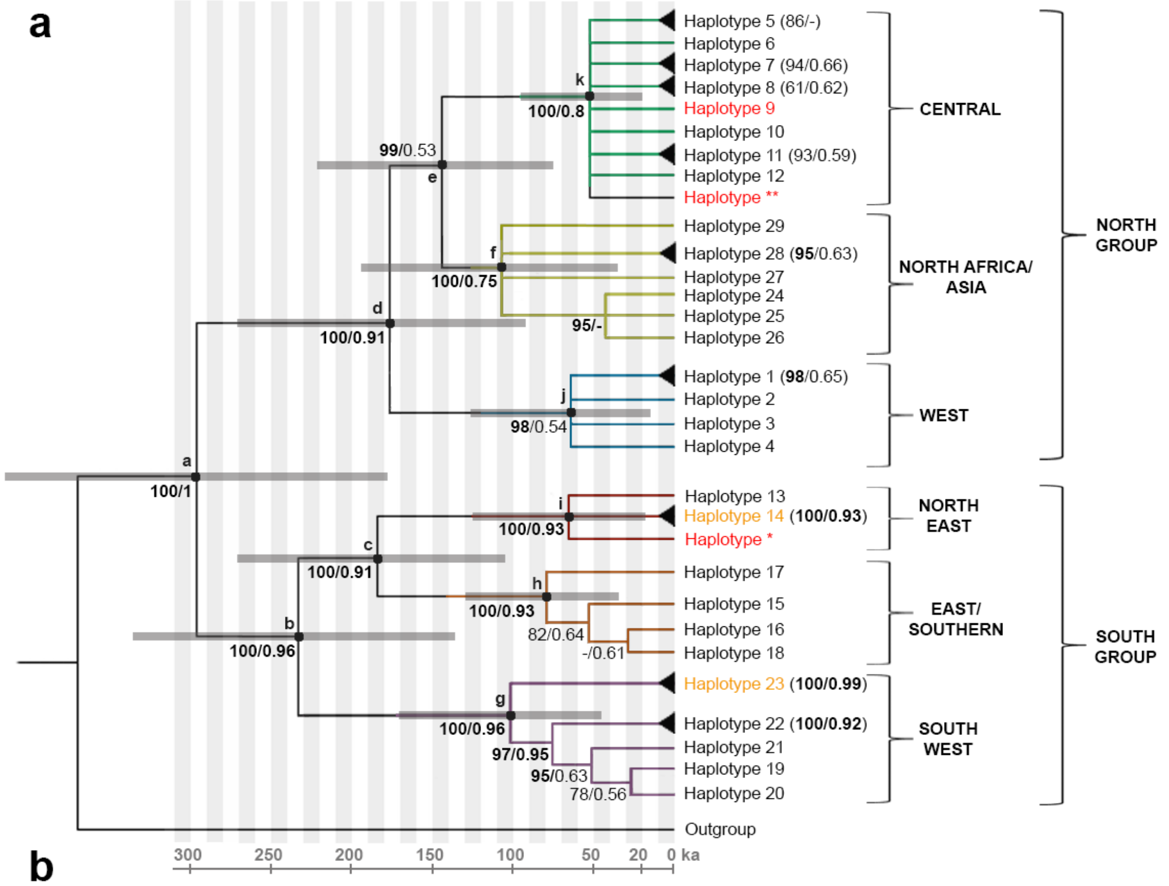

b

Clade	Node	Date (95% HPD)	Clade	Node	Date (95% HPD)	Clade	Node	Date (95% HPD)	Clade	Node	Date (95% HPD)
Modern Lion	a	293.8 kya (178.6-416.3)	North	d	176.8 kya (92.9-271.8)	South West Africa	g	100.4 kya (43.4-170.2)	West Africa	j	63.4 kya (15.0-127.6)
South	b	231.2 kya (138.7-337.2)	Central Africa - India	e	142.0 kya (75.3 - 222.8)	East/ Southern Africa	h	78.1 kya (34.8-129.7)	Central Africa	k	51.7 kya (19.7-95.0)
East/Southern - North East Africa	c	182.3 (104.3-271.4)	India - North Africa	f	106.4 kya (35.8-194.2)	North East Africa	i	65.1 kya (18.1-124.5)			

Supplemental Figure 2-1. Phylogenetic analyses for the complete lion dataset, including sixteen mitochondrial genomes and 178 cytb+ctrl reg. sequences. a) Phylogenetic tree of lion populations throughout their complete geographic range, based on complete mitochondrial genomes and cytB+ctrl reg. sequences. Branch colours correspond to haplotype colours in Supplemental Figure 2-2. Populations mentioned above as long branches with missing intermediate haplotypes, are indicated in orange. Populations with limited information regarding their origin, which were excluded from analyses presented in the main text, are shown in red. Support is indicated as posterior probability (Bayesian analysis)/bootstrap support (ML analysis). Branches with a single haplotype have been collapsed to improve readability. Support for these branches is indicated by a black triangle at the tip of the branch (support shown in the label). Nodes which have been included for divergence time estimates are indicated with letters and 95% HPD node bars. Distance to outgroup and nodes without dated split is not in proportion to divergence time. b) divergence time estimates and 95% HPD from BEAST analysis, also indicated as error bars in Supplemental Figure 2-1A.

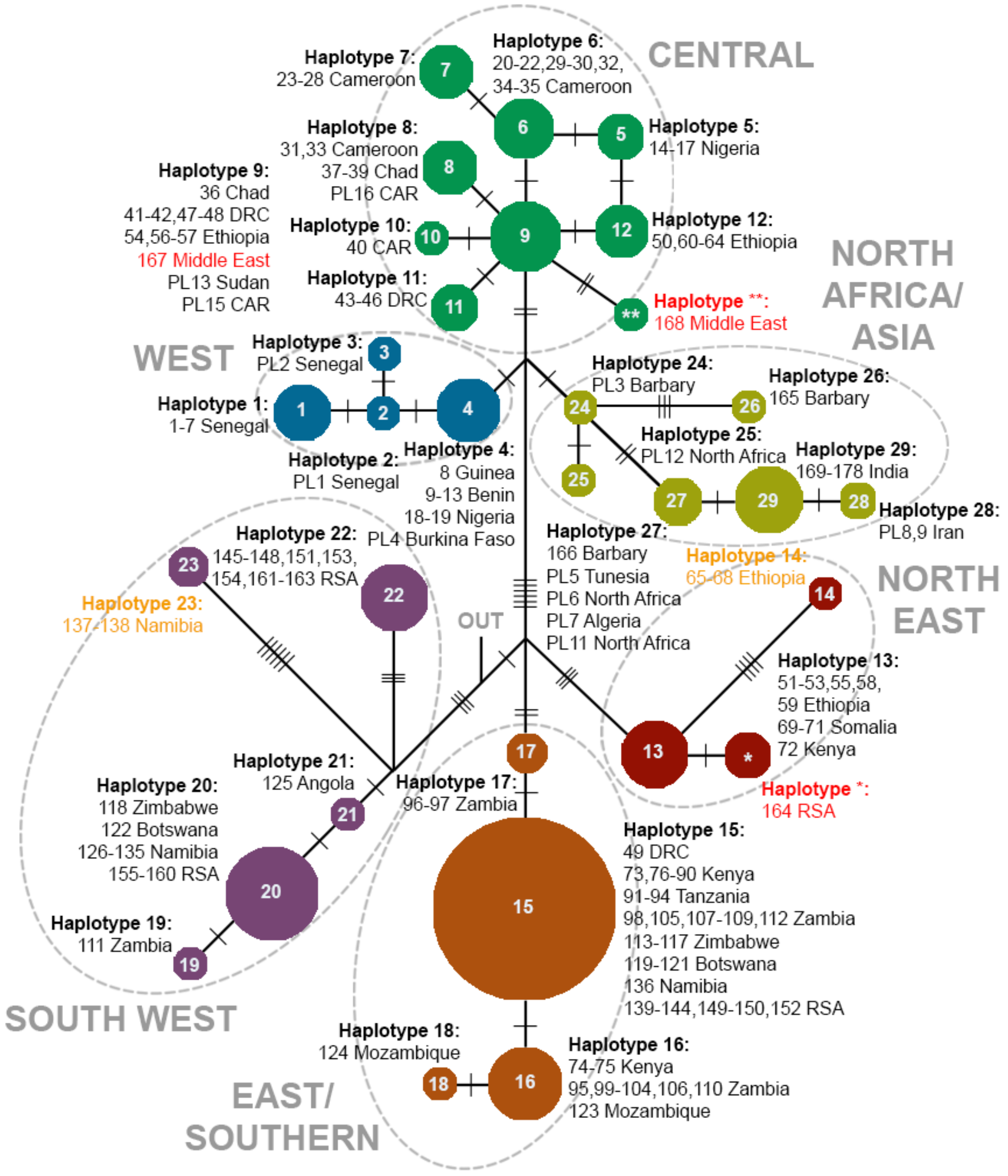

Supplemental Figure 2-2. Haplotype network based on cytB+ctrl reg. sequences of lions throughout their entire geographic range. Dashed lines indicate the groups discerned Bayesian/ML analysis in Supplemental Figure 2-1A. Populations indicated above as long branches with missing intermediate haplotypes, are shown in orange. Populations with limited information regarding their origin, which were excluded from analyses presented in the main text, are indicated in red. Haplotype size is proportional to its frequency in the dataset. Hatch marks represent a change in the DNA sequence. The connection to outgroup species is indicated by “OUT”.

References

1. Bruche, S. *et al.* A genetically distinct lion (Panthera leo) population from Ethiopia. *Eur. J. Wildl. Res.* **59,** 215–225 (2012).
2. Dubach, J. *et al.* Molecular genetic variation across the southern and eastern geographic ranges of the African lion, Panthera leo. *Conserv. Genet.* **6,** 15–24 (2005).
3. Bertola, L. D. *et al.* Genetic diversity, evolutionary history and implications for conservation of the lion (Panthera leo) in West and Central Africa. *J. Biogeogr.* **38,** 1356–1367 (2011).
4. Antunes, A. *et al.* The evolutionary dynamics of the lion Panthera leo revealed by host and viral population genomics. *PLoS Genet.* **4,** e1000251 (2008).
5. Dubach, J. M., Briggs, M. B., White, P. A., Ament, B. A. & Patterson, B. D. Genetic perspectives on 'Lion Conservation Units' in Eastern and Southern Africa. *Conserv. Genet.* **1942,** (2013).
6. Barnett, R. *et al.* Revealing the maternal demographic history of Panthera leo using ancient DNA and a spatially explicit genealogical analysis. *BMC Evol. Biol.* **14,** 70 (2014).
7. Steinmetz, A. *et al.* Lens-anomalies and other ophthalmic findings in a group of closely-related angola lions (Panthera leo bleyenberghi). *Zoo Biol.* **25,** 433–439 (2006).

Supplemental Information 2. Details on sample storage, processing and analysis.

Samples were preserved dried, in 95% ethanol or in buffer (0.15 M NaCl, 0.05 M Tris-HCl, 0.001 M EDTA, pH = 7.5) and stored at -20°C (Supplemental Table 4). For blood and tissue samples DNA was extracted using the DNeasy Blood & Tissue kit (Qiagen) following the manufacturer's protocol. For the scat and the museum samples, a protocol for aDNA extractions from bone and teeth [1] was followed. In all cases a mock extraction was included to check for contamination. All museum samples were processed in the aDNA facility of DNAmarkerpoint, Leiden University, which is physically isolated from other laboratories and where no previous work on felids had been conducted. In addition, two scat samples which contained strongly degraded DNA, 8.Guinea and 30.Cameroon, were included in the aDNA procedure. Before each extraction, the surfaces in the extraction room were cleaned using 10% bleach and all materials were cleaned and irradiated with UV light for a minimum of one hour.

The complete mitochondrial genome was analyzed for ten individuals by sequencing on an Illumina HiSeq2000 using 99 bp paired-end sequencing with 200-400 bp insert size (Leiden Genome Technology Center, Leiden, The Netherlands). In the first run, two individuals (9.Benin and 89.Kenya) were tagged and pooled with leopard DNA (ratios 1:1:2 for 9.Benin, 89.Kenya and 179.Leopard respectively). In the following two runs, four individuals (21.Cameroon+71.Somalia+162.RSA+ 174.India and 42.DRC+95.Zambia+96.Zambia+131.Namibia) were tagged and equimolarily pooled. Resulting reads were identified based on the unique adapter sequences.

For four individuals the complete mtDNA was analysed by performing two long range PCRs for amplifying all ~18,000 bp. Primers were designed based on known leopard sequences available on Genbank using Primer3v 0.4.0 [2]. Primer sites were chosen such that the forward and corresponding reverse primer were not both located in one of the known numts that have been identified in felids [3–5] (see below, Supplemental Figure 1-1). For amplification either the LA PCR kit (TaKaRa) or the GoTaq Long PCR Master Mix (Promega) was used (Supplemental Table 4). Resulting PCR products were cut out from the gel, cleaned with the Wizard SV gel and PCR Clean-Up kit (Promega) and sonically fragmented. Barcoded Libraries for sequencing were prepared from the fragmented PCR products using the Rapid Library Preparation Kit (Roche). Emulsion PCR and sequencing were performed on the 454/Roche FLX Genome Sequencer Titanium (Forensic Laboratory for DNA Research, Leiden, The Netherlands) according to the protocol.

Cytochrome B, tRNAThr, tRNAPro and the left domain of the control region (hereafter referred to as cytB+ctrl reg.) were amplified using three primer pairs in high quality blood and tissue samples, five primer pairs in the scat samples and twelve primer pairs in the aDNA samples. See Supplemental Table 5 for primer sequences. All primers were designed using the web-based software

Primer3v 0.4.0 [2]. The modern samples were amplified using Taq DNA Polymerase (Invitrogen) or Phire Hot Start II DNA Polymerase (Thermo Scientific), depending on the amplification success. Annealing temperature was adjusted according to primer pair and according to previous PCR results (for details see Supplemental Table 4). The museum samples were amplified using AmpliTaq Gold DNA Polymerase (Invitrogen) and following a half-nested approach: in the first round (40 cycles) primer aDNA1F was combined with primer aDNA2R and a 1:50 dilution of the PCR product was used as a template for a second round PCR (40 cycles), in which primer aDNA1F was combined with aDNA1R and primer aDNA2F was combined with aDNA2R etc. In all cases multiple negative PCR controls were included to check for contamination.

Sequencing of the short, non-aDNA PCR products was performed by Macrogen Inc., Amsterdam, The Netherlands. The aDNA samples were sequenced on the Roche/454 platform (Forensic Laboratory for DNA Research, Leiden, The Netherlands). The 12 PCR products for each museum sample were equimolarily pooled, and after a test run containing one sample, the remaining 17 samples were divided into two pools, which were analysed in two separate runs. To check for contamination and to distinguish the samples after sequencing, a unique combination of tags attached to the primers was used for each individual. In addition to the 454 sequencing, 22 PCR products were cloned to confirm sequences with a coverage <10 or inconclusive results (i.e. called base supported by <90% of available reads). Cloning was performed using the Invitrogen TOPO cloning kit following the manufacturer's protocol. From each cloned PCR product, between three and eight colonies were picked. Picked colonies were lysed by heating the cells in 30 µl of water for 10 minutes (min) at 95°C. Cell lysates were amplified with M13 primers using the following PCR: 2 µl $MgCl_2$ (25 mM), 2 µl 10× PCR buffer, 0.25 µl dNTPs (2.5 mM each), 0.24 µl Taq polymerase, 0.5 µl M13 primers (10 µM each), and 2 µl cell lysate, with water added to a final volume of 20 µl. The PCR program was: 94°C for 5 min followed by 40 cycles of 94°C for 30 seconds (s), 55°C for 45 s, 72°C for 45 s and a final extension step of 72°C for 4 min. The PCR products were sequenced by Macrogen Inc., Amsterdam, The Netherlands. Overlap between independent PCR products were used to check for DNA damage and sequencing errors. Unique point mutations (i.e. observed in a single sample) were checked by an independent PCR and sequencing for modern samples, or cloning for aDNA samples.

Read data from Illumina and 454 platforms were analysed using CLC Genomics (CLCBio). A leopard mitochondrial genome available on GenBank (EF551002.1) was used as reference. Mapping was performed by using default settings, except for length fraction and similarity fraction, which were increased to 0.8 and 0.85 respectively. Consensus sequences were extracted and aligned visually with Macrogen sequences. Since we observed one region that seemed to be absent in all Illumina samples, but present in all sequences derived by PCR and Sanger sequencing, and another

region where the opposite was true, we constructed a new reference sequence and repeated the mapping of all Illumina and 454 reads, which lead to a more consistent coverage across the reference sequence. Sequences covering cytB+ctrl reg. that had already been analysed in earlier publications [6–9] were added to the dataset for phylogenetic analyses.

Since Roche/454 sequencing does not perform well with mononucleotide repeats, all mononucleotide repeats of >3 bp were manually checked. Gaps resulting from inconclusive base calling were substituted by an ambiguous nucleotide. This was also done for inconclusive results on six positions in three aDNA samples which could not be resolved and a 62bp region with insufficient coverage in sample 165.Barbary. Two repetitive regions in the control region, RS-2 and RS-3, were excluded from the analysis, since aligning was difficult and the region is known to be heteroplasmic [10]. In addition, a mononucleotide repeat of cytosines of variable length was excluded due to unknown homology (bp 1382-1393 in cytB+ctrl reg.). For phylogenetic analysis 179.Leopard was used as an outgroup and supplemented by six sequences from Genbank: clouded leopard (*Neofelis nebulosa*: DQ257669.1), snow leopard (*Panthera uncia*: EF551004.1), two sequences of tiger (*Panthera tigris*: JF357968.1 (Bengal) and JF357974.1 (Amur)), one sequence of leopard (*Panthera pardus*: EF551002.1) and one sequence of cave lion (*Panthera leo spelaea*: KC701376.1 + DQ899901.1). In addition, two complete mitochondrial genomes from Asiatic lions were included (JQ904290.1 and KC834784.1) (not included in Figures). All sequences were manually aligned using Bioedit (v7.1.3.0)[11]. Since the sequences from Genbank did not align well in the control region, likely due to the assembly method, this region of the Genbank sequences was replaced by ambiquous nucleotides to eliminate the influence of assembly quality.

Although numts (nuclear copies of mtDNA) are well documented in felids, contamination of these non-mitochondrial sequences in the presented dataset is highly unlikely. All included lion sequences in the manuscript deviated strongly from previously published nuclear pseudogenes from lion and other cats [4,5,12]. In addition, all haplotypes presented in this manuscript align well to lions sequences from other studies [8,9,13–15]. Dubach et al. (2005) validated their sequences by checking a total of 18 clones of the cytochrome b gene in two individuals. Two PCR reactions were performed to verify authentic substitutions from those due to PCR artifact. The same approach was used by Barnett et al. (2006) for verification of the distinguished haplotypes. Since the observed pattern in sequence divergence between population is consistent with our expectations and previously published data, we are confident that numt contamination does not play a role in the presented data.

References

1. Rohland, N. & Hofreiter, M. Ancient DNA extraction from bones and teeth. *Nat. Protoc.* **2,** 1756–1762 (2007).
2. Rozen, S. & Skaletsky, H. J. in *Bioinforma. Methods Protoc. Methods Mol. Biol.* (S., K. & S., M.) 365–386 (Humana Press, 2000).
3. Lopez, J. V., Cevario, S. & O'Brien, S. J. Complete nucleotide sequences of the domestic cat (Felis catus) mitochondrial genome and a transposed mtDNA tandem repeat (Numt) in the nuclear genome. *Genomics* **33,** 229–46 (1996).
4. Cracraft, J., Feinstein, J., Vaughn, J. & Helm-Bychowski, K. Sorting out tigers (Panthera tigris): mitochondrial sequences, nuclear inserts, systematics, and conservation genetics. *Anim. Conserv.* **1,** 139–150 (1998).
5. Kim, J.-H. *et al.* Evolutionary analysis of a large mtDNA translocation (numt) into the nuclear genome of the Panthera genus species. *Brain, Behav. Immun.* **22,** 629–629 (2006).
6. Barnett, R., Yamaguchi, N., Barnes, I. & Cooper, A. Lost populations and preserving genetic diversity in the lion Panthera leo: Implications for its ex situ conservation. *Conserv. Genet.* **7,** 507–514 (2006).
7. Barnett, R., Yamaguchi, N., Barnes, I. & Cooper, A. The origin, current diversity and future conservation of the modern lion (Panthera leo). *Proc. R. Soc. B Biol. Sci.* **273,** 2119–2125 (2006).
8. Bertola, L. D. *et al.* Genetic diversity, evolutionary history and implications for conservation of the lion (Panthera leo) in West and Central Africa. *J. Biogeogr.* **38,** 1356–1367 (2011).
9. Bertola, L. D. *et al.* Autosomal and mtDNA Markers Affirm the Distinctiveness of Lions in West and Central Africa. *PLoS One* **10,** e0137975 (2015).
10. Jae-Heup, K., Eizirik, E., O'Brien, S. J. & Johnson, W. E. Structure and patterns of sequence variation in the mitochondrial DNA control region of the great cats. *Mitochondrion* **1,** 279–292 (2001).
11. Hall, T. BioEdit: a user-friendly biological sequence alignment editor and analysis program for Windows 95/98/NT. *Nucleic Acids Symp. Ser.* 95–98 (1999).
12. Janczewski, D. N., Modi, W. S., Stephens, J. C. & O'Brien, S. J. Mitochondrial 12S molecular evolution of mitochondrial 12S RNA and and cytochrome b sequences in pantherine lineage of Felidae. *Mol. Biol. Evol.* **12,** 690–707 (1995).
13. Barnett, R. *et al.* Revealing the maternal demographic history of Panthera leo using ancient DNA and a spatially explicit genealogical analysis. *BMC Evol. Biol.* **14,** 70 (2014).
14. Dubach, J. M., Briggs, M. B., White, P. A., Ament, B. A. & Patterson, B. D. Genetic perspectives on 'Lion Conservation Units' in Eastern and Southern Africa. *Conserv. Genet.* **1942,** (2013).
15. Dubach, J. *et al.* Molecular genetic variation across the southern and eastern geographic ranges of the African lion, Panthera leo. *Conserv. Genet.* **6,** 15–24 (2005).

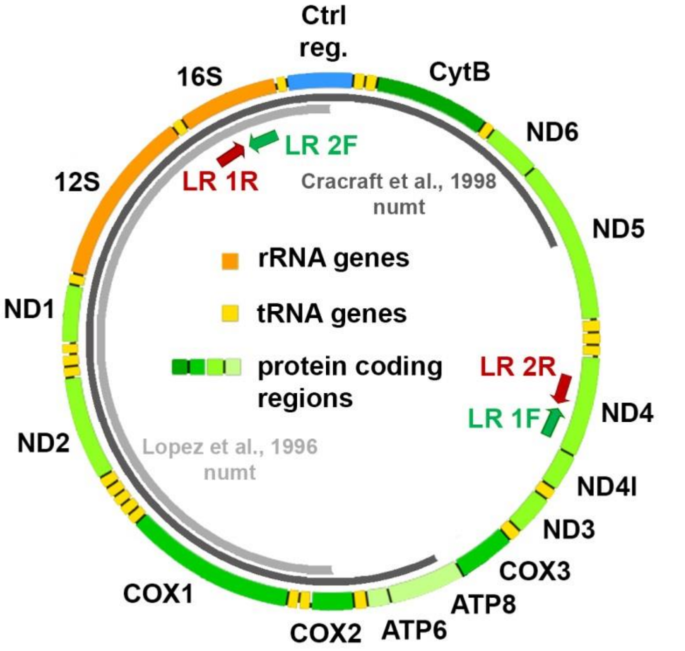

Supplemental Figure 1-1. Schematic representation of the mitochondrial genome (adjusted figure, source: http://commons.wikimedia.org/), with location of known numts and primer sites for long range PCR.

Supplemental Table 4. Overview of processing of lion samples included

Number	Country	Location	Sample type	Storage	DNA Extraction	PCR	Primer set	Sequencing	Genetic region	NCBI Accession***
1	Senegal	Niokolo-Koba NP	Scat	dried (silica)	Silica method	Protocol I	Set2	Macrogen	cytB+ctrl reg.	KJ652247
2	Senegal	Niokolo-Koba NP	Scat	dried (silica)	Silica method	Protocol II	Set2 / Set3	Macrogen	cytB+ctrl reg.	KJ652247
3	Senegal	Niokolo-Koba NP	Scat	dried (silica)	Silica method	Protocol I	Set2 / Set3	Macrogen	cytB+ctrl reg.	KJ652247
4	Senegal	Niokolo-Koba NP	Scat	dried (silica)	Silica method	Protocol I	Set2	Macrogen	cytB+ctrl reg.	KJ652247
5	Senegal	Niokolo-Koba NP	Scat	dried (silica)	Silica method	Protocol I	Set2 / Set3	Macrogen	cytB+ctrl reg.	KJ652247
6	Senegal	Niokolo-Koba NP	Scat	dried (silica)	Silica method	Protocol I	Set2 / Set3	Macrogen	cytB+ctrl reg.	KJ652247
7	Senegal	Niokolo-Koba NP	Scat	dried (silica)	Silica method	Protocol I	Set2 / Set3	Macrogen	cytB+ctrl reg.	KJ652247
8	Guinea	-	Scat	dried	Silica aDNA	Protocol III	Set3	454	cytB+ctrl reg.	DQ018996**
9	Benin	Pendjari NP	Blood	buffer	DNeasy	-	-	Illumina	complete mtDNA	KP001497
10	Benin	Pendjari NP	Blood	buffer	-	-	-	-	cytB+ctrl reg.	GU131165
11	Benin	Pendjari NP	Tissue	buffer	DNeasy	Protocol I	Set1	Macrogen	cytB+ctrl reg.	GU131165
12	Benin	Pendjari NP	Tissue	buffer	DNeasy	Protocol I	Set1	Macrogen	cytB+ctrl reg.	GU131165
13	Benin	Pendjari NP	Tissue	buffer	DNeasy	Protocol I	Set1	Macrogen	cytB+ctrl reg.	GU131165
14	Nigeria	Yankari GR	Scat	95% ethanol	Silica method	Protocol I	Set2	Macrogen	cytB+ctrl reg.	KM374706
15	Nigeria	Yankari GR	Scat	95% ethanol	Silica method	Protocol II	Set2	Macrogen	cytB+ctrl reg.	KM374706
16	Nigeria	Yankari GR	Scat	95% ethanol	Silica method	Protocol II	Set2	Macrogen	cytB+ctrl reg.	KM374706
17	Nigeria	Yankari GR	Scat	95% ethanol	Silica method	Protocol I	Set2	Macrogen	cytB+ctrl reg.	KM374706
18	Nigeria	Kainji NP	Scat	dried (silica)	Silica method	Protocol I	Set2	Macrogen	cytB+ctrl reg.	GU131165
19	Nigeria	Kainji NP	Scat	dried (silica)	Silica method	Protocol I	Set2	Macrogen	cytB+ctrl reg.	GU131165
20	Cameroon	Waza NP	Blood	buffer	DNeasy	Protocol I	Set1	Macrogen	cytB+ctrl reg.	GU131171
21	Cameroon	Waza NP	Blood	buffer	DNeasy	Protocol I	Set1	Illumina/Macrogen	complete mtDNA	KP001502
22	Cameroon	Waza NP	Blood	buffer	DNeasy	Protocol I	Set1	Macrogen	cytB+ctrl reg.	GU131173**
23	Cameroon	Waza NP	Blood	buffer	DNeasy	Protocol I	Set1	Macrogen	cytB+ctrl reg.	GU131174**
24	Cameroon	Waza NP	Blood	buffer	DNeasy	Protocol I	Set1	Macrogen	cytB+ctrl reg.	GU131175**
25	Cameroon	Waza NP	Tissue	buffer	DNeasy	Protocol I	Set1	Macrogen	cytB+ctrl reg.	AY781202
26	Cameroon	Waza NP	Tissue	buffer	DNeasy	Protocol I	Set1	Macrogen	cytB+ctrl reg.	AY781203
27	Cameroon	Waza NP	Tissue	buffer	DNeasy	Protocol I	Set1	Macrogen	cytB+ctrl reg.	AY781204
28	Cameroon	Waza NP	Tissue	buffer	DNeasy	Protocol I	Set1	Macrogen	cytB+ctrl reg.	AY781205
29	Cameroon	Faro NP	Blood	buffer	DNeasy	Protocol I	Set2 / Set3	Macrogen	cytB+ctrl reg.	GU131171
30	Cameroon	Faro NP	Scat	buffer	Silica aDNA	Protocol III	Set3	454	cytB+ctrl reg.	GU131171
31	Cameroon	Bénoué NP	Tissue	buffer	DNeasy	PROMEGA	LR	454/Macrogen	complete mtDNA	KP001493
32	Cameroon	Bénoué NP	Tissue	buffer	DNeasy	Protocol I	Set1	Macrogen	cytB+ctrl reg.	GU131170**
33	Cameroon	Bouba Njida NP	Scat	buffer	Silica method	Protocol II	Set2	Macrogen	cytB+ctrl reg.	AY781198
34	Cameroon	Bouba Njida NP	Scat	buffer	Silica method	Protocol II	Set2	Macrogen	cytB+ctrl reg.	GU131171
35	Cameroon	Bouba Njida NP	Scat	buffer	Silica method	Protocol II	Set2	Macrogen	cytB+ctrl reg.	GU131171
36	Chad	Zakouma NP	DNA	-	-	-	-	-	cytB+ctrl reg.	AY781200
37	Chad	Zakouma NP	DNA	-	-	-	-	-	cytB+ctrl reg.	AY781198
38	Chad	Zakouma NP	DNA	-	-	-	-	-	cytB+ctrl reg.	AY781199
39	Chad	Zakouma NP	DNA	-	-	-	-	-	cytB+ctrl reg.	AY781197
40	CAR	Birao	Bone	-	Silica aDNA	Protocol III	Set3	454	cytB+ctrl reg.	KM374705
41	DRC	Garamba NP	Tissue	dried	DNeasy	Protocol I	Set1	Macrogen	cytB+ctrl reg.	AY781200, DQ018993 - DQ018994
42	DRC	Garamba NP	Tissue	dried	DNeasy	Protocol I	Set1 / Set3	Illumina/Macrogen	complete mtDNA	KP001506
43	DRC	Garamba NP	Tissue	dried	DNeasy	Protocol I	Set1	Macrogen	cytB+ctrl reg.	KJ652248
44	DRC	Garamba NP	Tissue	dried	DNeasy	Protocol I	Set1	Macrogen	cytB+ctrl reg.	KJ652248
45	DRC	Garamba NP	Tissue	dried	DNeasy	Protocol I	Set1	Macrogen	cytB+ctrl reg.	KJ652248
46	DRC	Garamba NP	Tissue	dried	DNeasy	Protocol I	Set1 / Set3	Macrogen	cytB+ctrl reg.	KJ652248
47	DRC	-	DNA	-	-	TAKARA	LR	454/Macrogen	complete mtDNA	KP001494
48	DRC	-	DNA	-	-	-	-	Macrogen	cytB+ctrl reg.	DQ018994
49	DRC	Ruindi Plains S. of Lake Edward	Bone	-	Silica aDNA	Protocol III	Set3	454/Cloning	cytB+ctrl reg.	GU131166 - GU131168
50	Ethiopia	Gambela NP	Scat	buffer	Silica method	Protocol I	Set2	Macrogen	cytB+ctrl reg.	KJ652249
51	Ethiopia	Kaffa Province	Scat	buffer	Silica method	Protocol I	Set2	Macrogen	cytB+ctrl reg.	GU131181 - GU131182
52	Ethiopia	Nechisar NP	Scat	buffer	Silica method	Protocol I	Set2	Macrogen	cytB+ctrl reg.	GU131181 - GU131182
53	Ethiopia	Nechisar NP	Scat	buffer	Silica method	Protocol I	Set2	Macrogen	cytB+ctrl reg.	GU131181 - GU131182
54	Ethiopia	Bale Mountains NP	Scat	buffer	Silica method	Protocol II	Set2	Macrogen	cytB+ctrl reg.	AY781200
55	Ethiopia	Oromia region, Hudet	Scat	buffer	Silica method	Protocol I	Set2 / Set3	Macrogen	cytB+ctrl reg.	GU131181 - GU131182
56	Ethiopia	Somali region, Dolo Ado	Scat	buffer	Silica method	Protocol I	Set2	Macrogen	cytB+ctrl reg.	AY781200
57	Ethiopia	Somali region, Kebri Dehar	Scat	buffer	Silica method	Protocol I	Set2	Macrogen	cytB+ctrl reg.	AY781200
58	Ethiopia	Somali region	Scat	buffer	Silica method	Protocol I	Set2	Macrogen	cytB+ctrl reg.	GU131181 - GU131182
59	Ethiopia	Somali region	Scat	buffer	Silica method	Protocol I	Set2 / Set3	Macrogen	cytB+ctrl reg.	GU131181 - GU131182
60	Ethiopia	-	DNA	-	-	TAKARA	LR	454	complete mtDNA	KP001495
61	Ethiopia	-	DNA	-	-	Protocol I	Set1	Macrogen	cytB+ctrl reg.	KJ652249
62	Ethiopia	-	DNA	-	-	Protocol I	Set1	Macrogen	cytB+ctrl reg.	KJ652249
63	Ethiopia	-	DNA	-	-	Protocol I	Set1	Macrogen	cytB+ctrl reg.	KJ652249
64	Ethiopia	-	DNA	-	-	Protocol I	Set1	Macrogen	cytB+ctrl reg.	KJ652249
65	Ethiopia	-	Blood	buffer	-	-	-	-	cytB+ctrl reg.	AY781207
66	Ethiopia	-	Blood	buffer	-	-	-	-	cytB+ctrl reg.	AY781208
67	Ethiopia	-	Blood	buffer	-	-	-	-	cytB+ctrl reg.	AY781209
68	Ethiopia	-	Blood	buffer	-	-	-	-	cytB+ctrl reg.	AY781210
69	Somalia	-	Scat	buffer	-	-	-	-	cytB+ctrl reg.	GU131181
70	Somalia	-	Scat	buffer	-	-	-	-	cytB+ctrl reg.	GU131182
71	Somalia	-	Tissue	95% ethanol	DNeasy	-	-	Illumina	complete mtDNA	KP001499
72	Kenya	Aberdare NP	Tissue	95% ethanol	DNeasy	Protocol I	Set1	Macrogen	cytB+ctrl reg.	GU131181 - GU131182
73	Kenya	Maasai Mara NR	Scat	buffer	Silica method	Protocol II	Set2	Macrogen	cytB+ctrl reg.	GU131166 - GU131168
74	Kenya	Maasai Mara NR	Scat	buffer	Silica method	Protocol II	Set2	Macrogen	cytB+ctrl reg.	KJ652250
75	Kenya	Maasai Mara NR	Scat	buffer	Silica method	Protocol II	Set2 / Set3	Macrogen	cytB+ctrl reg.	KJ652250
76	Kenya	Maasai Mara NR	Scat	buffer	Silica method	Protocol II	Set2 / Set3	Macrogen	cytB+ctrl reg.	GU131166 - GU131168
77	Kenya	Maasai Mara NR	Scat	buffer	Silica method	Protocol I	Set2	Macrogen	cytB+ctrl reg.	GU131166 - GU131168
78	Kenya	Maasai Mara NR	Scat	buffer	Silica method	Protocol I	Set2	Macrogen	cytB+ctrl reg.	GU131166 - GU131168
79	Kenya	Amboseli NP	Tissue	buffer	DNeasy	Protocol I	Set1	Macrogen	cytB+ctrl reg.	GU131166 - GU131168
80	Kenya	Amboseli NP	Blood	buffer	DNeasy	Protocol I	Set1	Macrogen	cytB+ctrl reg.	GU131166 - GU131168
81	Kenya	Amboseli NP	Tissue	buffer	DNeasy	Protocol I	Set1	Macrogen	cytB+ctrl reg.	GU131166 - GU131168
82	Kenya	Amboseli NP	Blood	buffer	DNeasy	Protocol I	Set1	Macrogen	cytB+ctrl reg.	GU131166 - GU131168
83	Kenya	Amboseli NP	Blood	buffer	DNeasy	Protocol I	Set1 / Set3	Macrogen	cytB+ctrl reg.	GU131166 - GU131168
84	Kenya	Amboseli NP	Tissue	buffer	DNeasy	Protocol I	Set1 / Set3	Macrogen	cytB+ctrl reg.	GU131166 - GU131168
85	Kenya	Amboseli NP	Tissue	95% ethanol	DNeasy	Protocol I	Set1	Macrogen	cytB+ctrl reg.	GU131166 - GU131168
86	Kenya	Kuku group ranch	Hair	-	Silica method	Protocol I	Set1	Macrogen	cytB+ctrl reg.	GU131166 - GU131168
87	Kenya	Tsavo East NP	Scat	buffer	Silica method	Protocol I	Set2	Macrogen	cytB+ctrl reg.	GU131166 - GU131168
88	Kenya	Tsavo East NP	Scat	buffer	Silica method	Protocol I	Set2	Macrogen	cytB+ctrl reg.	GU131166 - GU131168
89	Kenya	Tsavo East NP	Tissue	95% ethanol	DNeasy	Protocol I	Set1	Illumina	complete mtDNA	KP001498
90	Kenya	Tsavo East NP	Tissue	95% ethanol	DNeasy	Protocol I	Set1	Macrogen	cytB+ctrl reg.	GU131166 - GU131168
91	Tanzania	Serengeti NP	DNA	-	-	Protocol II	Set2 / Set3	Macrogen	cytB+ctrl reg.	GU131166 - GU131168
92	Tanzania	Serengeti NP	DNA	-	-	Protocol II	Set2 / Set3	Macrogen	cytB+ctrl reg.	GU131166 - GU131168
93	Tanzania	Serengeti NP	DNA	-	-	Protocol I	Set2	Macrogen	cytB+ctrl reg.	GU131166 - GU131168
94	Tanzania	Ngorongoro Conservation Area	DNA	-	-	Protocol II	Set2 / Set3	Macrogen	cytB+ctrl reg.	GU131166 - GU131168
95	Zambia	Mpika town	Tissue	95% ethanol	DNeasy	Protocol I	Set1	Illumina/Macrogen	complete mtDNA	KP001503
96	Zambia	Mulobezi town	Tissue	95% ethanol	DNeasy	Protocol I	Set1	Illumina/Macrogen	complete mtDNA	KP001505
97	Zambia	Mumbwa town	Tissue	95% ethanol	DNeasy	Protocol I	Set1	Macrogen	cytB+ctrl reg.	KM374707
98	Zambia	north of Lusaka	Tissue	95% ethanol	DNeasy	Protocol I	Set1	Macrogen	cytB+ctrl reg.	GU131166 - GU131168
99	Zambia	Luangwa valley	Tissue	95% ethanol	DNeasy	Protocol I	Set1	Macrogen	cytB+ctrl reg.	KJ652250
100	Zambia	Luangwa valley	Tissue	95% ethanol	DNeasy	Protocol I	Set1	Macrogen	cytB+ctrl reg.	KJ652250
101	Zambia	Luangwa valley	Tissue	95% ethanol	DNeasy	Protocol I	Set1	Macrogen	cytB+ctrl reg.	KJ652250

102	Zambia	Luangwa valley	Tissue	95% ethanol	DNeasy	Protocol I	Set1	Macrogen	cytB+ctrl reg.	KJ652250
103	Zambia	Luangwa valley	Tissue	95% ethanol	DNeasy	Protocol I	Set1	Macrogen	cytB+ctrl reg.	KJ652250
104	Zambia	Luangwa valley	Tissue	95% ethanol	DNeasy	Protocol I	Set1	Macrogen	cytB+ctrl reg.	KJ652250
105	Zambia	Luangwa valley	Tissue	95% ethanol	DNeasy	Protocol I	Set1	Macrogen	cytB+ctrl reg.	GU131166 - GU131168
106	Zambia	Luangwa valley	Tissue	95% ethanol	DNeasy	Protocol I	Set1	Macrogen	cytB+ctrl reg.	KJ652250
107	Zambia	Luangwa valley	Tissue	95% ethanol	DNeasy	Protocol I	Set1	Macrogen	cytB+ctrl reg.	GU131166 - GU131168
108	Zambia	Luangwa valley	Tissue	95% ethanol	DNeasy	Protocol I	Set1	Macrogen	cytB+ctrl reg.	GU131166 - GU131168
109	Zambia	Luangwa valley	Tissue	95% ethanol	DNeasy	Protocol I	Set1	Macrogen	cytB+ctrl reg.	GU131166 - GU131168
110	Zambia	Luangwa valley	Tissue	95% ethanol	DNeasy	Protocol I	Set1	Macrogen	cytB+ctrl reg.	KJ652250
111	Zambia	Victoria Falls	Tissue	dried	Silica aDNA	Protocol III	Set3	454/Cloning	cytB+ctrl reg.	KM374702
112	Zambia	Mweru Wantipa	Bone	-	Silica aDNA	Protocol III	Set3	454/Cloning	cytB+ctrl reg.	GU131166 - GU131168
113	Zimbabwe	Save Valley Conservancy	DNA	-	-	Protocol II	Set2 / Set3	Macrogen	cytB+ctrl reg.	GU131166 - GU131168
114	Zimbabwe	Save Valley Conservancy	DNA	-	-	Protocol I	Set2 / Set3	Macrogen	cytB+ctrl reg.	GU131166 - GU131168
115	Zimbabwe	Save Valley Conservancy	DNA	-	-	Protocol I	Set1	Macrogen	cytB+ctrl reg.	GU131166 - GU131168
116	Zimbabwe	Save Valley Conservancy	DNA	-	-	Protocol I	Set2 / Set3	Macrogen	cytB+ctrl reg.	GU131166 - GU131168
117	Zimbabwe	Nuanetsi area	Bone	-	Silica aDNA	Protocol III	Set3	454	cytB+ctrl reg.	GU131166 - GU131168
118	Zimbabwe	Robin's Camp	Bone	-	Silica aDNA	Protocol III	Set3	454	cytB+ctrl reg.	KJ652251
119	Botswana	-	Scat	buffer	Silica method	Protocol I	Set2	Macrogen	cytB+ctrl reg.	GU131166
120	Botswana	-	Scat	buffer	Silica method	Protocol I	Set2 / Set3	Macrogen/Cloning	cytB+ctrl reg.	GU131167**
121	Botswana	-	Scat	buffer	Silica method	Protocol I	Set2	Macrogen	cytB+ctrl reg.	GU131168**
122	Botswana	Aha Hills	Tissue	dried	Silica aDNA	Protocol III	Set3	454	cytB+ctrl reg.	KJ652251
123	Mozambique	Caia	Bone	dried	Silica aDNA	Protocol III	Set3	454/Cloning	cytB+ctrl reg.	KJ652250
124	Mozambique	Changara	Tissue	dried	Silica aDNA	Protocol III	Set3	454	cytB+ctrl reg.	KM374703
125	Angola	-	Bone	dried	Silica aDNA	Protocol III	Set3	454	cytB+ctrl reg.	KM374704
126	Namibia	Etosha NP	DNA	-	-	Protocol II	Set2 / Set3	Macrogen	cytB+ctrl reg.	KJ652251
127	Namibia	Etosha NP	DNA	-	-	Protocol II	Set2	Macrogen	cytB+ctrl reg.	KJ652251
128	Namibia	Easter Caprivi	Blood	95% ethanol	DNeasy	Protocol I	Set1	Macrogen	cytB+ctrl reg.	KJ652251
129	Namibia	East Etosha	Blood	95% ethanol	DNeasy	Protocol II	Set1	Macrogen	cytB+ctrl reg.	KJ652251
130	Namibia	East and West Caprivi/Botswana	Blood	95% ethanol	DNeasy	Protocol II	Set1	Macrogen	cytB+ctrl reg.	KJ652251
131	Namibia	Eastern Etosha	Blood	95% ethanol	DNeasy	Protocol I	Set1	Illumina	complete mtDNA	KP001504
132	Namibia	Eastern Etosha	Blood	95% ethanol	DNeasy	Protocol II	Set1	Macrogen	cytB+ctrl reg.	KJ652251
133	Namibia	West Caprivi and Angola	Blood	95% ethanol	DNeasy	Protocol I	Set1	Macrogen	cytB+ctrl reg.	KJ652251
134	Namibia	Etosha Central	Blood	95% ethanol	DNeasy	Protocol I	Set1	Macrogen	cytB+ctrl reg.	KJ652251
135	Namibia	East Etosha	Blood	95% ethanol	DNeasy	Protocol I	Set1	Macrogen	cytB+ctrl reg.	KJ652251
136	Namibia	Erongo/Walvis Bay	Bone	-	Silica aDNA	Protocol III	Set3	454/Cloning	cytB+ctrl reg.	GU131166 - GU131168
137	Namibia	-	Blood	buffer	DNeasy	PROMEGA	LR	454	complete mtDNA	KP001496
138	Namibia	-	Blood	buffer	DNeasy	Protocol I	Set1	Macrogen	cytB+ctrl reg.	GU131180
139	RSA	Kalahari Gemsbok NP	DNA	-	-	Protocol II	Set1	Macrogen	cytB+ctrl reg.	GU131166 - GU131168
140	RSA	Kalahari Gemsbok NP	DNA	-	-	Protocol I	Set2 / Set3	Macrogen	cytB+ctrl reg.	GU131166 - GU131168
141	RSA	Kgalagadi to Tswalu	DNA	-	-	Protocol I	Set1 / Set3	Macrogen	cytB+ctrl reg.	GU131166 - GU131168
142	RSA	Kgalagadi to Tswalu	DNA	-	-	Protocol I	Set1 / Set3	Macrogen	cytB+ctrl reg.	GU131166 - GU131168
143	RSA	Kgalagadi Transfrontier Park	DNA	-	-	Protocol I	Set1 / Set3	Macrogen	cytB+ctrl reg.	GU131166 - GU131168
144	RSA	Kgalagadi Transfrontier Park	DNA	-	-	Protocol I	Set1 / Set3	Macrogen	cytB+ctrl reg.	GU131166 - GU131168
145	RSA	Kruger NP: Gogonthaba, Malelane	DNA	-	-	Protocol I	Set1 / Set3	Macrogen	cytB+ctrl reg.	GU131184
146	RSA	Kruger NP: Gogonthaba, Malelane	DNA	-	-	Protocol I	Set1 / Set3	Macrogen	cytB+ctrl reg.	GU131184
147	RSA	Kruger NP: Skukuza Phabeni/ Nwaswitshaka watergat pad junction	DNA	-	-	Protocol I	Set1 / Set3	Macrogen	cytB+ctrl reg.	GU131184
148	RSA	Kruger NP: Skukuza Phabeni/ Nwaswitshaka watergat pad junction	DNA	-	-	Protocol I	Set1 / Set3	Macrogen	cytB+ctrl reg.	GU131184
149	RSA	Kruger NP: Pretoriuskop, Fayi loop	DNA	-	-	Protocol I	Set1 / Set3	Macrogen	cytB+ctrl reg.	GU131166 - GU131168
150	RSA	Kruger NP: Lower Sabie, S128	DNA	-	-	Protocol I	Set1 / Set3	Macrogen	cytB+ctrl reg.	GU131166 - GU131168
151	RSA	Kruger NP: Crocodile Bridge	DNA	-	-	Protocol I	Set1 / Set3	Macrogen	cytB+ctrl reg.	GU131184
152	RSA	Kruger NP: Crocodile Bridge	DNA	-	-	Protocol I	Set1 / Set3	Macrogen	cytB+ctrl reg.	GU131166 - GU131168
153	RSA	Kruger NP: Stolznek, North of Biyamiti	DNA	-	-	Protocol I	Set1 / Set3	Macrogen	cytB+ctrl reg.	GU131184
154	RSA	Kruger NP: Stolznek	DNA	-	-	Protocol I	Set1	Macrogen	cytB+ctrl reg.	GU131184
155	RSA	Venetia-Limpopo NR, Tuli Block	DNA	-	-	Protocol I	Set1	Macrogen	cytB+ctrl reg.	KJ652251
156	RSA	Venetia-Limpopo NR, Tuli Block	DNA	-	-	Protocol I	Set1	Macrogen	cytB+ctrl reg.	KJ652251
157	RSA	Venetia-Limpopo NR, Tuli Block	DNA	-	-	Protocol I	Set1	Macrogen	cytB+ctrl reg.	KJ652251
158	RSA	Venetia-Limpopo NR, Tuli Block	DNA	-	-	Protocol II	Set1	Macrogen	cytB+ctrl reg.	KJ652251
159	RSA	Venetia-Limpopo NR, Tuli Block	DNA	-	-	Protocol I	Set1	Macrogen	cytB+ctrl reg.	KJ652251
160	RSA	Venetia-Limpopo NR, Tuli Block	DNA	-	-	Protocol II	Set1	Macrogen	cytB+ctrl reg.	KJ652251
161	RSA	Kruger NP: Timbavati	Blood	buffer	DNeasy	Protocol I	Set1	Macrogen	cytB+ctrl reg.	GU131183**
162	RSA	Kruger NP: Timbavati	Blood	buffer	DNeasy	Protocol I	Set1	Illumina	complete mtDNA	KP001500
163	RSA	Kruger NP: Timbavati	Blood	buffer	DNeasy	Protocol I	Set1	Macrogen	cytB+ctrl reg.	GU131185**
164*	RSA	-	Bone	-	Silica aDNA	Protocol III	Set3	454/Cloning	cytB+ctrl reg.	not uploaded
165	Barbary	-	Bone	-	Silica aDNA	Protocol III	Set3	454/Cloning	cytB+ctrl reg.	KM374700
166	Barbary	-	Tissue (footpad)	dried	Silica aDNA	Protocol III	Set3	454	cytB+ctrl reg.	KM374701
167*	Middle-East	-	Bone	-	Silica aDNA	Protocol III	Set3	454/Cloning	cytB+ctrl reg.	AY781200
168*	Middle-East	-	Bone	-	Silica aDNA	Protocol III	Set3	454/Cloning	cytB+ctrl reg.	not uploaded
169	India	Gir Forest NP	DNA	-	-	Protocol I	Set1 / Set3	Macrogen	cytB+ctrl reg.	GU131176
170	India	Gir Forest NP	Tissue	95% ethanol	DNeasy	Protocol I	Set1 / Set3	Macrogen	cytB+ctrl reg.	GU131176
171	India	Gir Forest NP	DNA	-	-	Protocol I	Set1 / Set3	Macrogen	cytB+ctrl reg.	GU131176
172	India	Gir Forest NP	DNA	-	-	Protocol I	Set1 / Set3	Macrogen	cytB+ctrl reg.	GU131176
173	India	Gir Forest NP	DNA	-	-	Protocol I	Set1 / Set3	Macrogen	cytB+ctrl reg.	GU131176
174	India	Gir Forest NP	DNA	-	-	Protocol I	Set1	Illumina	complete mtDNA	KP001501
175	India	Gir Forest NP	Scat	buffer	Silica method	Protocol I	Set2	Macrogen	cytB+ctrl reg.	GU131176
176	India	Gir Forest NP	Scat	buffer	Silica method	Protocol I	Set2	Macrogen	cytB+ctrl reg.	GU131177
177	India	Gir Forest NP	Scat	buffer	Silica method	Protocol I	Set2	Macrogen	cytB+ctrl reg.	GU131178**
178	India	Gir Forest NP	Scat	buffer	Silica method	Protocol I	Set2	Macrogen	cytB+ctrl reg.	AY781206
179	Senegal (PL1)	-	-	-	-	-	-	GenBank	cytB+ctrl reg.	KJ545522.1 + DQ248048.1
180	Senegal (PL2)	-	-	-	-	-	-	GenBank	cytB+ctrl reg.	KJ545523.1 + DQ248048.1
181	Barbary (PL3)	-	-	-	-	-	-	GenBank	cytB+ctrl reg.	KJ545524.1 + DQ248055.1
182	Burkina Faso (PL4)	-	-	-	-	-	-	GenBank	cytB+ctrl reg.	KJ545525.1 + DQ248047.1
183	Tunisia (PL5)	-	-	-	-	-	-	GenBank	cytB+ctrl reg.	KJ545526.1 + DQ248055.1
184	North Africa (PL6)	-	-	-	-	-	-	GenBank	cytB+ctrl reg.	KJ545527.1 + DQ248055.1
185	Algeria (PL7)	-	-	-	-	-	-	GenBank	cytB+ctrl reg.	KJ545528.1 + DQ248055.1
186	Iran (PL8)	-	-	-	-	-	-	GenBank	cytB+ctrl reg.	KJ545529.1 + DQ248054.1
187	Iran (PL9)	-	-	-	-	-	-	GenBank	cytB+ctrl reg.	KJ545530.1 + DQ248054.1
188	North Africa "Tower of London" (PL11)	-	-	-	-	-	-	GenBank	cytB+ctrl reg.	KJ545531.1 + DQ248055.1
189	North Africa "Tower of London" (PL12)	-	-	-	-	-	-	GenBank	cytB+ctrl reg.	KJ545532.1 + DQ248055.1
190	Sudan (PL13)	-	-	-	-	-	-	GenBank	cytB+ctrl reg.	KJ545533.1 + DQ248052.1
191	CAR (PL15)	-	-	-	-	-	-	GenBank	cytB+ctrl reg.	KJ545534.1 + DQ248050.1
192	CAR (PL16)	-	-	-	-	-	-	GenBank	cytB+ctrl reg.	KJ545535.1 + DQ248050.1
193	Asiatic lion (*Panthera leo persica*)	India	-	-	-	-	-	GenBank	complete mtDNA	JQ904290.1
194	Asiatic lion (Panthera leo persica)	India	-	-	-	-	-	GenBank	complete mtDNA	KC834784.1
195	Cave lion (*Panthera leo spelaea*)	Germany	-	-	-	-	-	GenBank	cytB+ctrl reg.	KC701376.1 + DQ899901.1

196	Leopard (*Panthera pardus*)	Amur	blood	buffer	Dneasy kit	Protocol I	Set1	Illumina	complete mtDNA	KP001507
197	Leopard (*Panthera pardus*)	Unknown	-	-	-	-	-	GenBank	complete mtDNA	EF551002.1
198	Tiger (*Panthera tigris*)	Bengal	-	-	-	-	-	GenBank	complete mtDNA	JF357967.1
199	Tiger (*Panthera tigris*)	Bengal	-	-	-	-	-	GenBank	complete mtDNA	JF357968.1
200	Tiger (*Panthera tigris*)	Sumatra	-	-	-	-	-	GenBank	complete mtDNA	JF357969.1
201	Tiger (*Panthera tigris*)	Sumatra	-	-	-	-	-	GenBank	complete mtDNA	JF357970.1
202	Tiger (*Panthera tigris*)	Amur	-	-	-	-	-	GenBank	complete mtDNA	JF357973.1
203	Tiger (*Panthera tigris*)	Amur	-	-	-	-	-	GenBank	complete mtDNA	JF357974.1
204	Snow leopard (*Panthera uncia*)	Unknown	-	-	-	-	-	GenBank	complete mtDNA	EF551004.1
205	Clouded leopard (*Neofelis nebulosa*)	Unknown	-	-	-	-	-	GenBank	complete mtDNA	DQ257669.1

* Excluded from analyses presented in the main text. See Supplemental Information 2 for background information and additional analyses.
** Updated Genbank entry (see Bertola *et al.* , 2014)
*** Haplotypes which were already available in Genbank (e.g. from another sample location), have not been added again

PCR : **Protocol I**

Chemical	Volume (µl)
10x PCR buffer	2
2.5mM dNTPs	1.6
DNA template	1
10 µM primer F	0.8
10 µM primer R	0.8
25 mM MgCl2	0.8
10mg/ml BSA	0.8
5U/µl Taq polymerase	0.4
H2O	11.8
Total	20

PCR program		Temperature °C	Time (Minutes)
step 1		94	4:00
40 cycles of	step 2	94	0:20
	step 3	50-55	1:00
	step 4	72	1:00
step 5		72	10:00

Protocol II

Chemical	Volume (µl)
5x PCR Phire buffer	4
2.5mM dNTPs	0.4
DNA template	1
10 µM primer F	0.8
10 µM primer R	0.8
5U/µl Phire Taq polymerase	0.4
H2O	12.6
Total	20

PCR program		Temperature °C	Time (Minutes)
step 1		98	0:30
35 cycles of	step 2	98	0:05
	step 3	50-55	0:05
	step 4	72	1:00
step 5		72	1:00

Protocol III

Chemical	Volume (µl)
10x Amplitaq Gold buffer	1.5
2.5mM dNTPs	0.375
DNA template	3.75
10 µM primer F	1,125
10 µM primer R	1,125
25 mM MgCl2	2.4
5U/µl Taq polymerase	0.4
H2O	2,925
Total	15

PCR program		Temperature °C	Time (Minutes)
step 1		94	10:00
40 cycles of	step 2	94	0:30
	step 3	50	1:00
	step 4	72	1:00
step 5		72	4:00

TAKARA

Chemical	Volume (µl)
10x PCR buffer	2
2.5mM dNTPs	3.2
DNA template	0.8
20 µM primer F	0.545
20 µM primer R	0.545
25 mM MgCl2	2
5U/µl TAKARA Taq polymerase	0.2
H2O	10.71
Total	20

PCR program		Temperature °C	Time (Minutes)
step 1		94	1:00
40 cycles of	step 2	98	0:10
	step 3	56	1:00
	step 4	72	10:00
step 5		72	10:00

PROMEGA

Chemical	Volume (µl)
GoTaq Mix	10
DNA template	3
20 µM primer F	0.4
20 µM primer R	0.4
H2O	6.2
Total	20

PCR program		Temperature °C	Time (Minutes)
step 1		94	2:00
40 cycles of	step 2	94	0:30
	step 3	56	0:30
	step 4	65	9:00
step 5		72	10:00

Supplemental Table 5. Primers used in this study.

Primer Set1	3 Primers		
**Primer name**	**Annealing Temp**	**Sequence (5'-3')**	**Reference**
1F	50-55 ˚C	CGTTGTACTTCAACTATAAGAACTT	Bertola et al., 2011
1R	50-55 ˚C	ATGGGATTGCTGATAGGAGATTAG	Bertola et al., 2011
2F	53-55 ˚C	GTGGGGCCAAATATCCTTTT	Bertola et al., 2011
4R	53-55 ˚C	TTTTTGGTTTACAAGACCAAGGTA	Bertola et al., 2011
5F	53-55 ˚C	AAATCGCCTCCTCAAATGAA	Bertola et al., 2011
5R	53-55 ˚C	AATATTCATGGGAGGGCAGTC	Bertola et al., 2015

Primer Set2	5 Primers		
**Primer name**	**Annealing Temp**	**Sequence (5'-3')**	**Reference**
1F	50-55 ˚C	CGTTGTACTTCAACTATAAGAACTT	Bertola et al., 2011
1R	50-55 ˚C	ATGGGATTGCTGATAGGAGATTAG	Bertola et al., 2011
2F	51-53 ˚C	GTGGGGCCAAATATCCTTTT	Bertola et al., 2011
2R	51-53 ˚C	GAAGGCCTAGGATATCTTTGATTG	Bertola et al., 2015
3F	51-53 ˚C	GACTCAGATAAAATTCCATTCCA	Bertola et al., 2015
3R	51-53 ˚C	CATTATTCCTCGCTGTTTGG	Bertola et al., 2015
4F	51-53 ˚C	CAATTATCCCTGCCCTCCA	Bertola et al., 2015
4R	51-53 ˚C	TTTTTGGTTTACAAGACCAAGGTA	Bertola et al., 2011
5F	53-55 ˚C	AAATCGCCTCCTCAAATGAA	Bertola et al., 2011
5R	53-55 ˚C	AATATTCATGGGAGGGCAGTC	Bertola et al., 2015

Primer Set3	12 Primers		
**Primer name**	**Annealing Temp**	**Sequence (5'-3')**	**Reference**
aDNA1F	50 ˚C	CGTTGTACTTCAACTATAAGAACTT	this study
aDNA1R	50 ˚C	CTAGAAAGAGGCCGGTGAGAA	this study
aDNA2F	50 ˚C	GCTCCTTATTAGGAGTATGCTTAATCC	this study
aDNA2R	50 ˚C	CATGCATGTATAGGCAGATAAAGA	this study
aDNA3F	50 ˚C	TGGCTGAATTATCCGGTACCTA	this study
aDNA3R	50 ˚C	GCACCTCAAAAGGATATTTGG	this study
aDNA4F	50 ˚C	AGCTACAGCCTTCATAGGATATGT	this study
aDNA4R	50 ˚C	TGGAAGGATGAAGTGGAAGG	this study
aDNA5F	50 ˚C	GGAGGCTTCTCAGTAGACAAAG	this study
aDNA5R	50 ˚C	TGATTGTATAGTATGGATGGAATGG	this study
aDNA6F	50 ˚C	CCCCTCAGGAATGGTATCTG	this study
aDNA6R	50 ˚C	ATATGGGGAGGGGTGCTTAG	this study
aDNA7F	50 ˚C	CTCACCAGACCTATTAGGAGATCC	this study
aDNA7R	50 ˚C	GAGGGCAGGGATAATTGCTA	this study
aDNA8F	50 ˚C	GCAATCCTCCGATCTATTCC	this study
aDNA8R	50 ˚C	CCAATTCATGTCAGGGTCAG	this study
aDNA9F	50 ˚C	CTTATTCTGATTCCTAGTAGCGGA	this study
aDNA9R	50 ˚C	CGTTCTCCTTTTTTGGTTTACAAG	this study
aDNA10F	50 ˚C	GCCTCCTCAAATGAAGAGTCT	this study
aDNA10R	50 ˚C	TGCAATATATGAATTGTGAAAGTTACG	this study
aDNA11F	50 ˚C	GCACCCAAAGCTGAAATTCT	this study
aDNA11R	50 ˚C	TCACTTGCTTTTCGTGGGG	this study
aDNA12F	50 ˚C	CTGTGCTTGCCCAGTATGTC	this study
aDNA12R	50 ˚C	CTGTACATGCTTAATATTCATGGG	this study

Primer Set LR	4 Primers		
**Primer name**	**Annealing Temp**	**Sequence (5'-3')**	**Reference**
LR 1F (NADH4)	56 ˚C	CTCACTTTCTGCACCTCTACTAGTCTTA	this study
LR 1R (16S)	56 ˚C	ACGGATCAGAAGTAAGAGACAGTAAAG	this study
LR 2F (16S)	56 ˚C	CATCACCTCTAGCATTTCCAGTATTAG	this study
LR 2R (NADH4)	56 ˚C	ACTAGCCATGAGCATTAGTGGTAGG	this study
